# Highly selective acid-catalyzed olefin isomerization of limonene to terpinolene by kinetic suppression of overreactions in a confined space of porous metal–macrocycle frameworks†

**DOI:** 10.1039/d2sc01561g

**Published:** 2022-06-03

**Authors:** Wei He, Shohei Tashiro, Mitsuhiko Shionoya

**Affiliations:** Department of Chemistry, Graduate School of Science, The University of Tokyo Tokyo 113-0033 Japan shionoya@chem.s.u-tokyo.ac.jp

## Abstract

Natural enzymes control the intrinsic reactivity of chemical reactions in the natural environment, giving only the necessary products. In recent years, challenging research on the reactivity control of terpenes with structural diversity using artificial host compounds that mimic such enzymatic reactions has been actively pursued. A typical example is the acid-catalyzed olefin isomerization of (+)-limonene, which generally gives a complex mixture due to over-isomerization to thermodynamically favored isomers. Herein we report a highly controlled conversion of (+)-limonene by kinetic suppression of over-isomerization in a confined space of a porous metal–macrocycle framework (MMF) equipped with a Brønsted acid catalyst. The terminal double bond of (+)-limonene migrated to one neighbor, preferentially producing terpinolene. This reaction selectivity was in stark contrast to the homogeneous acid-catalyzed reaction in bulk solution and to previously reported catalytic reactions. X-ray structural analysis and examination of the reaction with adsorption inhibitors suggest that the reactive substrates may bind non-covalently to specific positions in the confined space of the MMF, thereby inhibiting the over-isomerization reaction. The nanospaces of the MMF with substrate binding ability are expected to enable highly selective synthesis of a variety of terpene compounds.

## Abstract 2

A porous metal–macrocycle framework (MMF) equipped with a Brønsted acid catalyst in nanochannels enables highly selective isomerization of limonene to terpinolene by kinetically suppressing over-isomerization at confined acid sites.

## Graphical abstract

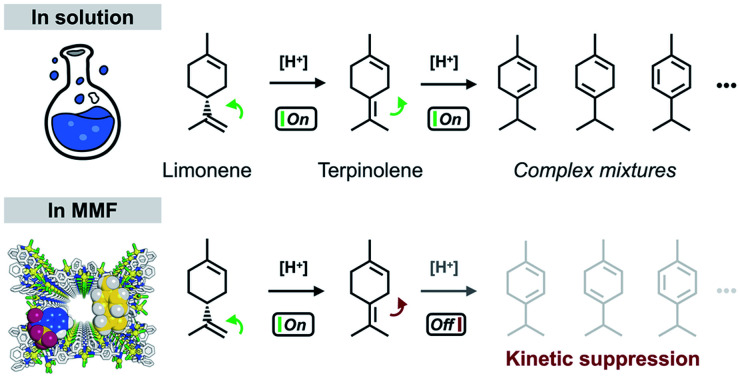

## Introduction

Natural enzymes are deeply involved in the synthesis of molecules necessary for life and in the formation and maintenance of their metabolic pathways by forming isolated spaces with precisely arranged substrate activation centers and by highly efficient and highly selective reactions specific to these spaces in the mild environment of nature.^1^ The reaction mode of the enzyme, which efficiently alters the intrinsic chemical reactivity of the substrate in the cavity based on thermodynamics and kinetics to produce the desired metabolites under mild conditions, may be the best exemplar for the construction of artificial enzymes. One of the most important biological reactions controlled by enzymes is the synthesis of terpenes. Terpenes are a group of natural products with very diverse structures synthesized from a limited number of poly-isoprene skeletons,^2–4^ and the control of these chemical reactions is an important issue in the field of catalytic chemistry.^5,6^ For instance, in the transformation reactions of terpenes, the position and conformation of the cationic intermediates are strictly regulated in the enzyme cavity to control the reaction.^7–9^

**Fig. 1:**
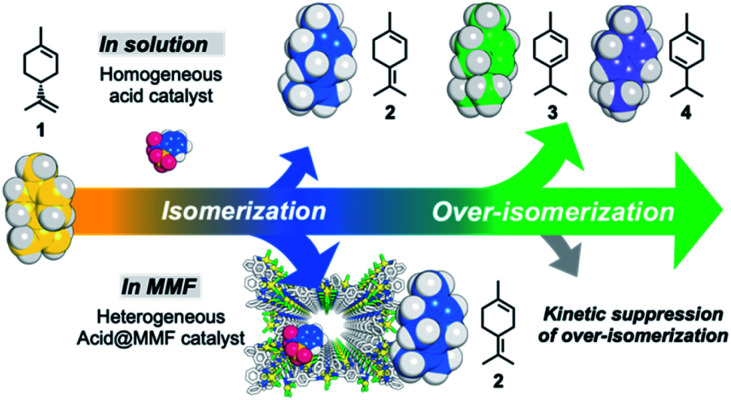
Acid-catalyzed (+)-limonene isomerization in bulk solution and in the MMF.

Inspired by the control of reactions in enzyme pockets in living organisms, in recent years there has been much research on the development of enzyme-like artificial host compounds that realize highly efficient and selective reactions of terpenes.^10–14^ However, it is very difficult to control the successive isomerization reactions of terpenes under thermodynamic control by external factors. (+)-Limonene (1), the main component of essential oils obtained from citrus fruits,^15^ such as orange, lemon, and grapefruit, is a typical example of monoterpene (C_10_H_16_). It was once concluded that the acid-catalyzed isomerization of limonene is an unselective process because it generally results in over-isomerization and gives a mixture of thermodynamically favorable products (Fig. 1).^16^ On the other hand, selective olefin migration using organometallic catalysts have attracted much attention,^17–24^ but examples of exploration using terpenes are still limited.^25^

Here we report the highly selective isomerization of the double bond of the side chain of (+)-limonene (1) to terpinolene (2) catalyzed by a Brønsted acid supported in the pores of a metal–macrocycle framework (MMF). This was achieved by kinetically suppressing the over-isomerization to α-terpinene (3) and γ-terpinene (4) and the subsequent oxidation to *p*-cymene (5), which generally takes place in homogeneous catalytic reactions based on thermodynamic control (Fig. 1). Acid-catalyzed limonene isomerization with the MMF showed 91% selectivity for 2, which proved to be the highest level of selectivity for this reaction. X-ray structural analysis and examination of the effects of the addition of adsorption inhibitors, (−)-α-pinene (6), (−)-β-pinene (7) and benzene (8), suggested that non-covalent molecular binding in the confined space of the MMF may be involved in the control of the limonene isomerization reaction.

**Fig. 2:**
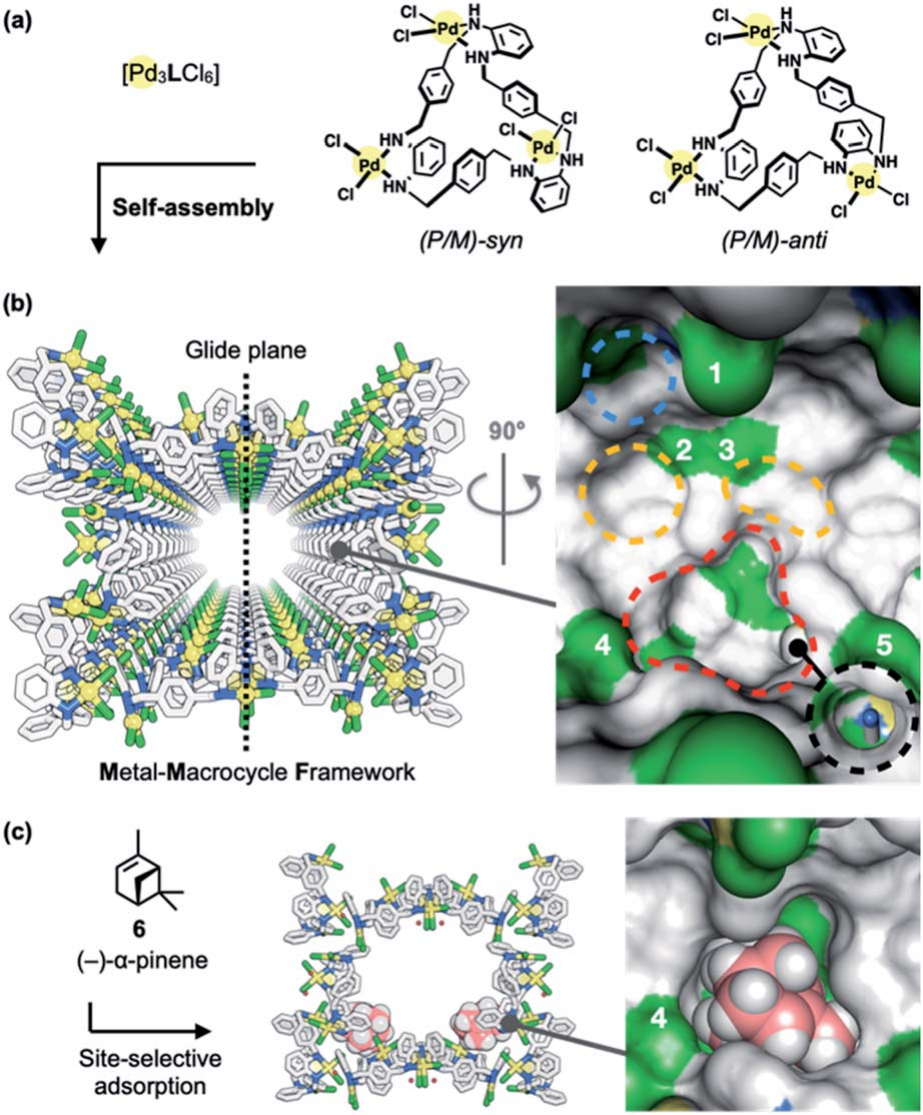
Metal–macrocycle framework (MMF). (a) Self-assembly of asymmetrically twisted Pd^II^-macrocycles into (b) a porous crystal MMF (sticks model) with five enantiomeric pairs of binding pockets (surface model). (c) Previously reported site-selective adsorption of (−)-α-pinene (6) (space-filling model) on the channel surface of the MMF.^30^ Blue, yellow, red, or black dashed circles indicate the ceiling-, side-, bottom-, or tubular-pockets of the MMF, respectively. MMF: Pd, yellow; Cl, green; N, blue; C, grey. 6: C, pink; H, white. Hydrogen atoms attached to the MMF were omitted for clarity. Green or blue surface represents exposed Cl or N–H groups of the MMF, respectively.

Porous MMFs have been shown to have a nanochannel structure formed by the self-assembly of four stereoisomers of Pd_3_LCl_6_ macrocycles [L = tris(*o*-phenylenediamine)] (Fig. 2a), with five enantiomeric pairs of well-defined binding pockets in their single-crystalline channels (Fig. 2b). Single crystal X-ray diffraction (ScXRD) analysis revealed that site-selective molecular adsorption in MMFs is possible through non-covalent interactions.^26–29^ For instance, a natural monoterpene, (−)-α-pinene (6), was site-selectively adsorbed to the bottom pockets of a MMF (Fig. 2c),^30^ and several terpenoids (terpene derivatives containing oxygen in their functional groups) were also recognized *via* hydrogen bonding.^31^ In addition, the substrate-specific cyclization of terpenoids was realized using a heterogeneous supramolecular acid catalyst, *p*-TsOH@MMF (*p*-TsOH = *p*-toluenesulfonic acid)^32^ with *p*-TsOH·H_2_O anchored to the channel surface.^31^ However, *p*-TsOH@MMF had no effect on the isomerization reaction of (+)-limonene (1). In order to find a more efficient acid catalyst, several strong acids were examined, and it was found that 2-nitrobenzenesulfonic acid (2-NBSA) stably immobilized to a MMF exhibited excellent reactivity for (+)-limonene isomerization.^33–35^

## Results and discussion

### Preparation of a supramolecular 2-NBSA@MMF catalyst

**Fig. 3:**
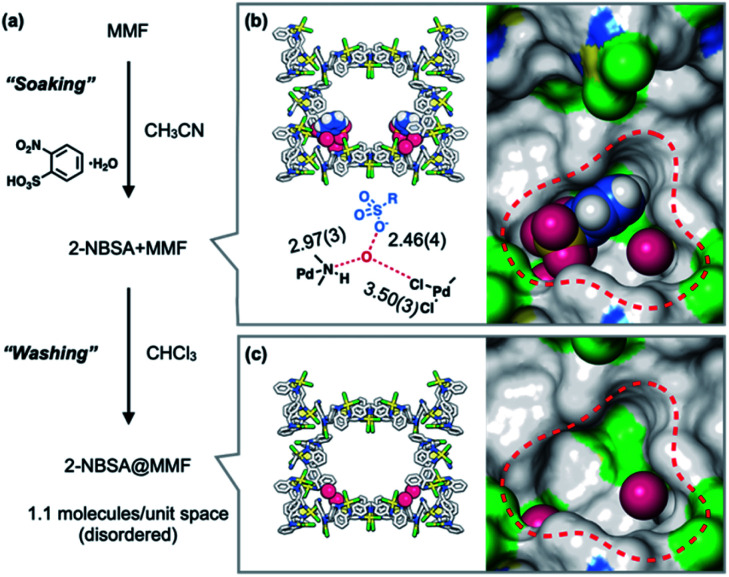
Immobilization of 2-NBSA·H_2_O in the MMF. (a) Schematic representation of the soaking and washing procedures. (b and c) Crystal structures after each step. MMF: sticks model or surface model; 2-NBSA and water: space-filling model. Hydrogen bonds are indicated by red dashed straight lines and adjacent values indicate distances (Å) between non-hydrogen atoms. Red dashed circles indicate the bottom pocket of the MMF. MMF: Pd, yellow; Cl, green; N, blue; C, grey. 2-NBSA and water molecules: O, red; C, blue; H, white. Hydrogen atoms attached to the MMF were omitted for clarity. Green or blue surface represents exposed Cl or N–H groups of the MMF, respectively.

The heterogeneous acid catalyst, 2-NBSA@MMF, was prepared by soaking MMF crystals in an acetonitrile solution of 2-NBSA·H_2_O for 1 day (Fig. 3a). The incorporation of 2-NBSA was confirmed by ScXRD. The results showed that 2-NBSA was site-selectively adsorbed to the bottom pockets of the MMF with 37% occupancy (Fig. 3b), accompanied by two water molecules. The sulfonate group formed a strong hydrogen bond with one of the water molecules with a short O⋯O distance (2.46 Å), which may be due to the salt bridge between R–SO_3_^−^ and H_3_O^+^.^36–38^^1^H NMR analysis of a solution of the crystals digested with DMSO-DCl before washing showed that an average of 2.8 molecules of 2-NBSA were non-covalently immobilized in the unit space (half of the unit cell) of the MMF. Next, ^1^H NMR analysis of a similar solution of 2-NBSA@MMF, in which the crystals were washed with CHCl_3_ until no 2-NBSA eluted into the supernatant, showed that an average of 1.1 molecules of 2-NBSA remained within the unit space of the MMF. ScXRD analysis after washing showed that the 2-NBSA molecules were highly disordered and the water molecules remained in the same binding positions (Fig. 3c). This suggests that 2-nitrobenzenesulfonate exists in disorder around the corner pockets with its counterion, H_3_O^+^, adsorbed to the pore surface.

### Acid-catalyzed olefin isomerization of (+)-limonene 1

**Fig. 4:**
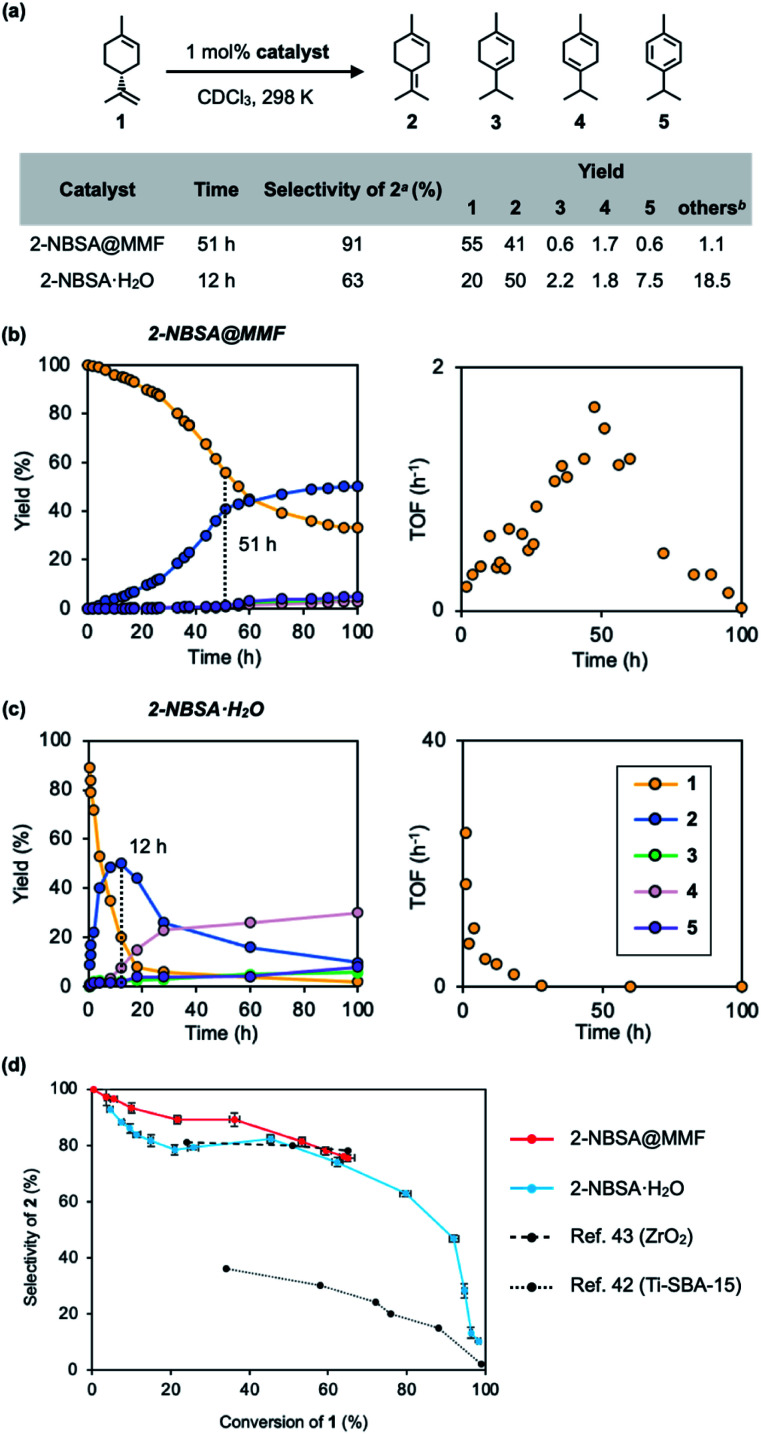
Isomerization of (+)-limonene (1) catalyzed by 2-NBSA@MMF or 2-NBSA·H_2_O. (a) Reaction scheme, conditions, and the results of reactions. (b and c) Time-course analysis (left) and time-rate (TOF: turnover frequency) plots (right) of both reactions. (d) Plot of conversion *vs.* selectivity for each catalyst. ^*a*^ The selectivity for 2 is defined as [2]/([2]+[3]+[4]+[5]+[others]); ^*b*^ The yields of “others”^39,40^ = 100% – (the total yields of 2–5 and the ratio of unreacted 1). The vertical and horizontal error bars in (d) represent the standard errors of selectivity and conversion ratio at each reaction time, based on three replicates, respectively (Fig. S16†).

Next, the isomerization reaction of (+)-limonene (1) was performed using 2-NBSA@MMF. As a result, 2-NBSA@MMF showed high reactivity for the isomerization reactions of 1, unlike the previous *p*-TsOH@MMF.^33^ The isomerization of 1 at 25 °C using 2-NBSA@MMF as the catalyst (1 mol% 2-NBSA, 0.91 mol% unit space of the MMF) produced 2 with 91% selectivity after 51 h (conversion rate of 1, 45%). When 3 mol% of 2-NBSA@MMF was used, the conversion of 1 increased to 85%, but the selectivity decreased to 48% (Fig. S12†). The heterogeneity of the 2-NBSA@MMF catalyst was also confirmed (Fig. S7 and S13†). In contrast, the isomerization catalyzed by 2-NBSA·H_2_O (1 mol%) in CDCl_3_ at 25 °C gave 2 with 63% selectivity after 12 h (at 80% conversion of 1) (Fig. 4a) (for the definition of “selectivity”,^16,39–43^ see the caption of Fig. 4). The reaction profiles showed that the over-isomerization to 3, 4 and 5 was more significantly suppressed when 2-NBSA@MMF was used as the catalyst (Fig. 4b), compared to the isomerization catalyzed by 2-NBSA·H_2_O (Fig. 4c). When compared at 100 h after the start of the reactions, the selectivity was reduced to 75% (at 67% conversion) in the case of the heterogeneous reaction using 2-NBSA@MMF due to a slight increase in over-isomerization during this time (Fig. 4b), while in the case of the homogeneous reaction using 2-NBSA·H_2_O, the selectivity decreased dramatically to 10% selectivity (at 98% conversion). This low selectivity was thought to be due to the consumption of 2 between 12 and 100 h (Fig. 4c). This was comparable to the overreactions reported in the literature,^16,42^ giving thermodynamically more favorable products such as 3, 4 (ref. 39 and 40) and 5. The selectivity of the catalytic isomerization of limonene (1) to 2 reported so far is 77% for TiO_2_/SiO_2_ supported phosphoric acid catalysts,^44^ 78% for ZrO_2_ catalysts,^43^ and 30% for mesoporous titanium catalysts.^42^ The plot of conversion *vs.* selectivity for each catalyst (Fig. 4d) shows that 2-NBSA@MMF is significantly more selective than the other catalysts up to about 50% conversion, but becomes as selective as 2-NBSA·H_2_O as the conversion further increases. Thus, to the best of our knowledge, 2-NBSA@MMF is one of the best acid catalysts in terms of selectivity for limonene isomerization reaction.

The time–rate plot of the isomerization reaction of 1 using 2-NBSA@MMF (Fig. 4b) shows an increase in the rate from 1 h to 50 h compared to the reaction under homogeneous catalytic conditions (Fig. 4c). This unique rate variation closely resembles the phenomenon in natural enzyme reactions in which chemical reactions are inhibited by the binding of substrates to the active center.^45,46^ Here, we propose that the over-isomerization of 2 is kinetically suppressed by the binding of 1 to the pore surface of the MMF. This hypothesis is consistent with the fact that the selectivity of 2 decreases as 1 is consumed (Fig. 4d).

**Fig. 5:**
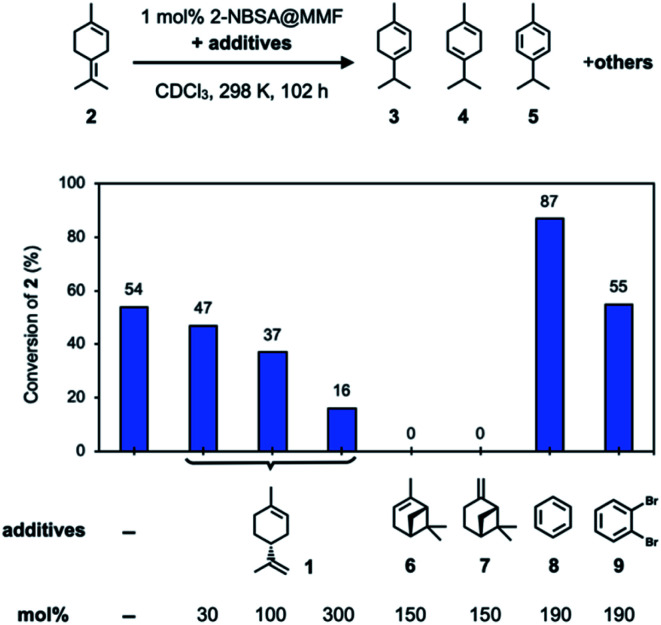
Investigation of the inhibitory effects of additives on the isomerization reaction of 2 using 2-NBSA@MMF at 25 °C for 102 h.

To confirm the inhibitory effect, we examined several additives that could inhibit the isomerization of 2 using 2-NBSA@MMF (Fig. 5). First, the isomerization reaction of 2 was carried out using 2-NBSA@MMF (1 mol% 2-NBSA). As a result, after 102 h at 25 °C, 54% of 2 was converted to a complex mixture containing 3 (5%), 4 (11%), 5 (14%) and other products. This result suggests that 2 is not necessarily the most thermodynamically stable isomer in the MMF. Next, the effects of several additives on the isomerization of 2 catalyzed by 2-NBSA@MMF were investigated. In the presence of 2-NBSA@MMF (1 mol% 2-NBSA), as the amount of (+)-limonene (1) added to 2 was increased from 30 mol%, 100 mol%, and 300 mol%, the conversion rate of 2 at 25 °C decreased to 47%, 37%, and 16% conversion of 2, respectively, after 102 h, and the isomerization of 2 was efficiently suppressed. Moreover, when 150 mol% of (−)-α-pinene (6) or (−)-β-pinene (7) was added, the isomerization of 2 was completely inhibited and the conversion of 2 was less than 1% under the same conditions. On the other hand, the addition of 190 mol% benzene (8) or 1,2-dibromobenzene (9), which binds to macrocycles on the channel surface^26^ (Fig. S28†), to 2 did not inhibit the isomerization of 2, and 87% or 55% of 2 was converted to the isomers or other products, respectively. The increase in conversion with the addition of 8 may be due to a cooperative effect of 8, which affects the arrangement of substrates and catalysts on the pore surface to change the reactivity. Such cooperative or competitive effects in the co-adsorption of multiple guests in the MMF have already been observed in our previous study.^28^ Limonene was also present as a product with or without the addition of 8 or 9 (Fig. S17a and S19†), suggesting that the interconversion of limonene and terpinolene (2) is reversible. Specifically, in the reaction without additives, limonene and 2 were obtained after 102 h at 298 K in 8 and 46% yields, respectively, which is almost comparable to those of the homogeneous reaction with 2-NBSA·H_2_O (limonene and 2 in 7 and 32% yields, respectively), suggesting that the MMF has little effect on the equilibrium of limonene and 2.

**Fig. 6:**
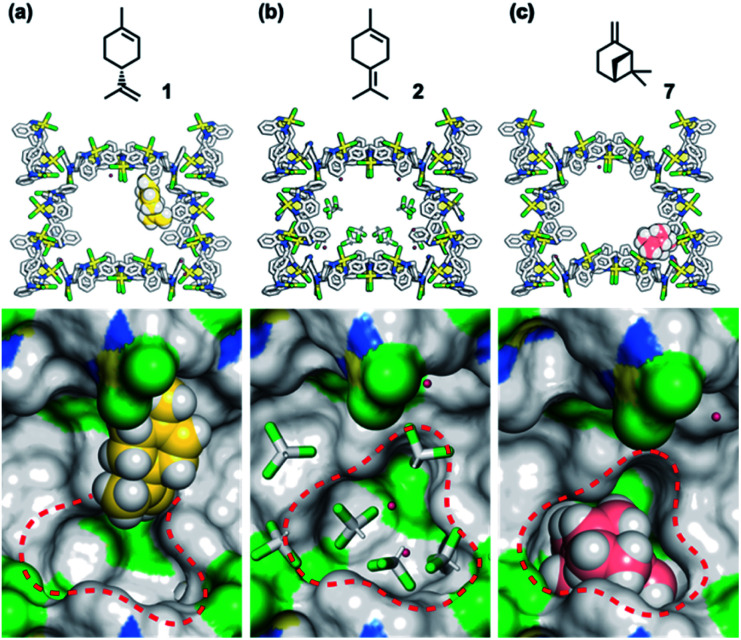
Crystallographic study of MMFs soaked in (a) a CHCl_3_ solution containing 1 (1.0 M), (b) a CHCl_3_ solution containing 2 (1.0 M), and (c) a CH_3_CN solution containing 7 (1.0 M). MMF: stick model or surface model; 1 and 7: space-filling model; water and CHCl_3_: stick model. Red dashed circles indicate the bottom pocket of the MMF. MMF: Pd, yellow; Cl, green; N, blue; C, grey. 1: C, yellow; H, white. 7: C, pink; H, white. Water and CHCl_3_: O, red; H, white; C, grey; Cl, green. Hydrogen atoms attached to the MMF were omitted for clarity. Green and blue surface represents exposed Cl and N–H groups of the MMF, respectively.

To understand the inhibitory effects observed in the MMF, the adsorption structures of 1, 2, and 7 on the MMF were analyzed by ScXRD. MMF crystals were soaked in a CHCl_3_ solution of (+)-limonene (1) at 25 °C for 1 day and then ScXRD analysis was performed at −180 °C. The crystal structure revealed that (+)-limonene was site-selectively adsorbed on the side pockets of the MMF with 60% occupancy (Fig. 6a). In the binding structure, the terminal olefin of 1 was oriented inside the bottom pockets of the MMF, as clearly supported by the electron density map (Fig. S22†). Therefore, the bottom pocket was partially blocked by 1. The analysis of the non-covalent interactions^47,48^ revealed van der Waals contacts between 1 and the three adjacent macrocycles (Fig. S23†). The space group changed from the MMF prototype, the centrosymmetric *P*2_1_/*c*, to the non-centrosymmetric *P*2_1_, with Flack^49^ and Hooft^50^ parameter values of 0.245(15) and −0.061(8), respectively. However, when MMF crystals were soaked in a CHCl_3_ solution of 2 under the same conditions, CHCl_3_ molecules, but not 2, were observed in the bottom pockets of the MMF (Fig. 6b), and the centrosymmetric *P*2_1_/*c* space group was maintained. On the other hand, soaking of MMF crystals in a CH_3_CN solution of 7 at 25 °C for 1 day resulted in the site-selective adsorption structure of 7 to the bottom pockets of the MMF with 91% occupancy (Fig. 6c). In this case, the framework of the MMF was particularly distorted, which could be attributed to the efficient non-covalent interactions between 7 and the five adjacent macrocycles (Fig. S27†). As a result, the space group changed to *P*2_1_, and the value of the Flack and Hooft parameters was 0.23(3) and −0.048(8), respectively. The above Flack parameters are presumably to be the result of incomplete guest occupancy and/or incomplete chirality transfer from the guest to the host.^29,49,51^

**Fig. 7:**
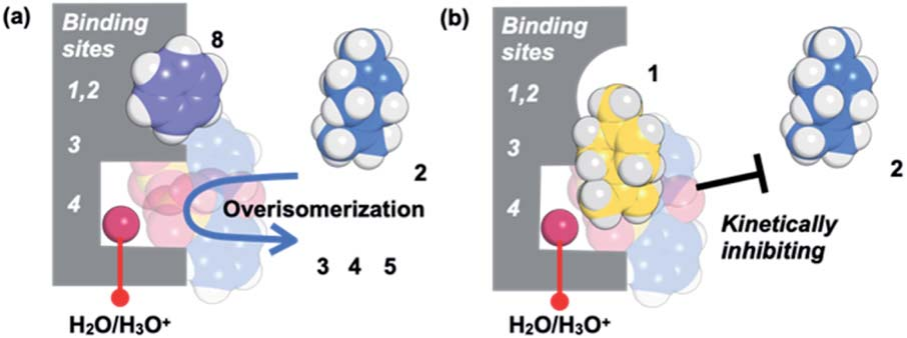
A possible mechanism that kinetically suppresses over-isomerization inside the MMF by sterically blocking the acid sites that are housed deep inside.

Based on the above guest adsorption structures, we discussed the reason why the over-isomerization of 2 is significantly suppressed in the MMF. Although it was difficult to determine the location of the acid sites in the MMF during the reaction, we can assume that the active H_3_O^+^ possibly stays in the bottom pocket (Fig. 7) as suggested by the crystal structure (Fig. 3). If the assumption is correct, the access of terpene substrates to the confined acid sites may be sterically obstructed by other terpenes (1, 6 and 7) that prefer binding to the bottom pocket and by 2-nitrobenzenesulfonate that seems to localize around H_3_O^+^ (Fig. 7b). This mechanism is consistent with the inhibitory experiments in which the addition of 1, 6 or 7 significantly slowed the isomerization of 2 into thermodynamically more stable 3 and 5. Moreover, the self-inhibition effect shown in Fig. 4b can also be explained by this hypothesis. On the other hand, the molecular adsorption in the MMF is complex and competitive,^28^ so that the adsorption of 1 is interfered with by other products, resulting in a reduction in selectivity to the same extent as that of 2-NBSA·H_2_O at 50% conversion (1/product molar ratio = 1 : 1), in marked contrast to the initial reaction (1/product molar ratio = 1 : 0.11 at 10% conversion) (Fig. 4d). Although the molecular recognition ability of the different binding pockets on the channel surface of the MMFs needs to be investigated in more detail, some of the effects described above may be important factors in the progression of the highly controlled isomerization of (+)-limonene (1) in the MMF.

### Acid-catalyzed cyclization of nerol (10) in a MMF

**Fig. 8:**
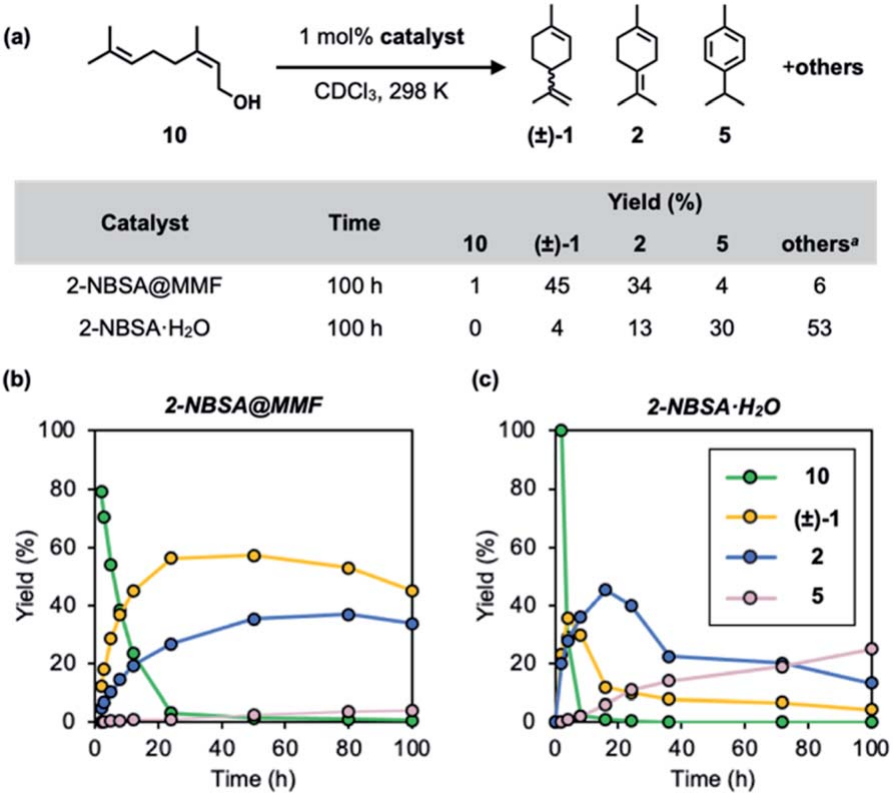
Acid-catalyzed cyclization of 10 catalyzed by 2-NBSA@MMF or 2-NBSA·H_2_O. (a) Reaction scheme, conditions, and the results of reactions. (b and c) Time-course analysis of both reactions. ^*a*^ The yields of “others” = 100% – (the total yields of 1, 2, and 5 and the ratio of unreacted 10).

Acid-catalyzed cyclization of nerol (10), a linear monoterpenoid, generally produces complex mixtures due to the difficulty in controlling the olefin isomerization of the cyclic product. Finally, the MMF catalyst was applied to this reaction in the hope that the overreaction would be suppressed in the confined space. As a result, it was confirmed that limonene and terpinolene were the major products in the MMF, and the over-isomerization reaction was significantly inhibited. Specifically, in the conversion of nerol (10) using 2-NBSA@MMF (1 mol% 2-NBSA) as an acid catalyst, limonene and terpinolene were selectively produced in 45% and 34% yields, respectively. The reaction profiles (Fig. 8a and b) appear to be different from those in Fig. 4b because of the direct formation of 2 from 10. The presence of 10 in the early stages of the reaction may affect the position of H_3_O^+^ and/or sulfonic acid in the MMF to alter the catalytic activity. In contrast, in the homogeneous reaction catalyzed by 2-NBSA·H_2_O (1 mol%) in CDCl_3_, the overreaction proceeded rapidly, yielding only 4% and 13% limonene and terpinolene, respectively, under the same conditions (Fig. 8). The results would pave the way for the control of the complex transformation of terpenes, including acid@MMF-catalyzed olefin isomerization processes.

## Conclusions

In conclusion, a supramolecular acid catalyst localized in the confined space of porous MMF crystals allowed highly selective isomerization from (+)-limonene (1) to terpinolene (2), and the selectivity is significantly higher than those of conventional catalysts. The high selectivity was achieved by suppressed over-isomerization from 2 to thermodynamically more favorable products. Crystal structure analyses suggest that the inhibitory effect is probably due to the confined environment of the acid moiety immobilized on the MMF. Highly controlled terpene conversion reactions are often seen in enzymatic reactions, giving products with specific pharmacological activities. Therefore, this reaction would provide a new artificial host-mediated enzyme-mimicry,^52–55^ which may lead to late-stage synthetic methods for non-natural derivatives of terpenes and complex molecules.

## Data availability

All of the relevant experimental data are available in the ESI.† Crystallographic data for MMFs have been deposited at the CCDC under 2133386–2133390.

## Author contributions

All authors designed the project, analyzed the results, and prepared the manuscript. W. H. performed all experimental studies.

## Conflicts of interest

The authors declare no competing financial interest.

## Supplementary Material

SC-013-D2SC01561G-s001

SC-013-D2SC01561G-s002
