# Correction: Highly selective acid-catalyzed olefin isomerization of limonene to terpinolene by kinetic suppression of overreactions in a confined space of porous metal–macrocycle frameworks

**DOI:** 10.1039/d2sc90161g

**Published:** 2022-08-11

**Authors:** Wei He, Shohei Tashiro, Mitsuhiko Shionoya

**Affiliations:** Department of Chemistry, Graduate School of Science, The University of Tokyo Tokyo 113-0033 Japan shionoya@chem.s.u-tokyo.ac.jp

## Abstract

Correction for ‘Highly selective acid-catalyzed olefin isomerization of limonene to terpinolene by kinetic suppression of overreactions in a confined space of porous metal–macrocycle frameworks’ by Wei He *et al.*, *Chem. Sci.*, 2022, **13**, 8752–8758, https://doi.org/10.1039/d2sc01561g.

The authors regret that there were errors in Fig. 2, Fig. 5 and Fig. 6 in the original article and Fig. S18 of the ESI. The stereochemistry of the chemical structural formulas for (−)-α-pinene (6) and (−)-β-pinene (7) was incorrectly reversed. The correct versions of the figures are shown below, and in the updated version of the ESI.

**Fig. 2:**
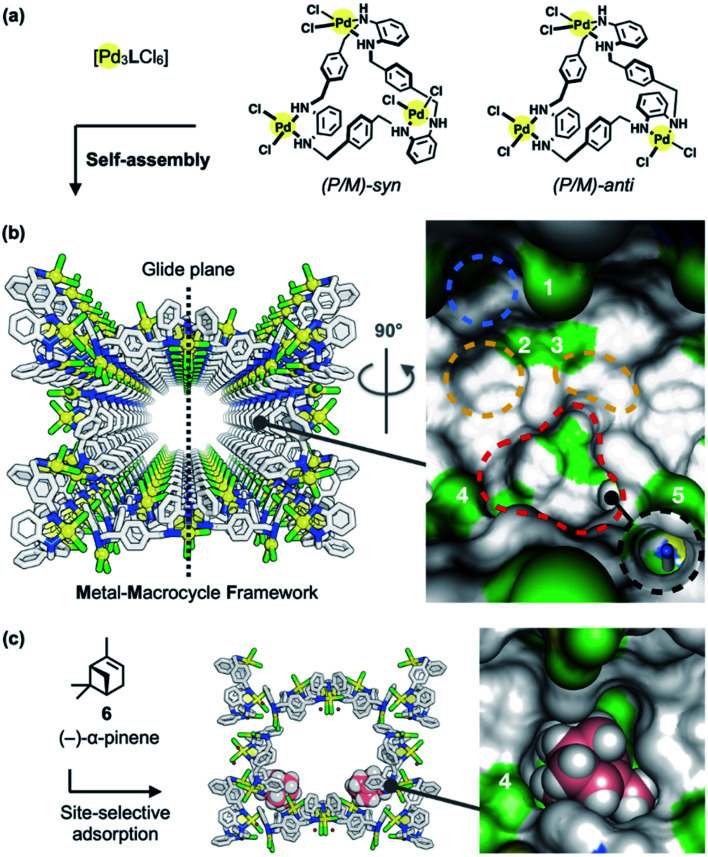
Metal–macrocycle framework (MMF). (a) Self-assembly of asymmetrically twisted Pd^II^-macrocycles into (b) a porous crystal MMF (sticks model) with five enantiomeric pairs of binding pockets (surface model). (c) Previously reported site-selective adsorption of (−)-α-pinene (6) (space-filling model) on the channel surface of the MMF.^1^ Blue, yellow, red, or black dashed circles indicate the ceiling-, side-, bottom-, or tubular-pockets of the MMF, respectively. MMF: Pd, yellow; Cl, green; N, blue; C, grey. 6: C, pink; H, white. Hydrogen atoms attached to the MMF were omitted for clarity. Green or blue surface represents exposed Cl or N–H groups of the MMF, respectively.

**Fig. 5:**
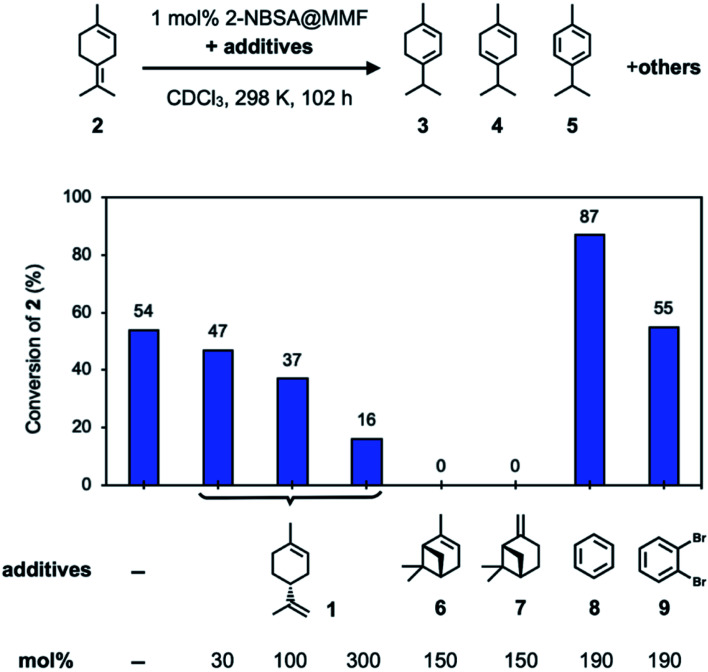
Investigation of the inhibitory effects of additives on the isomerization reaction of 2 using 2-NBSA@MMF at 25 °C for 102 h.

**Fig. 6:**
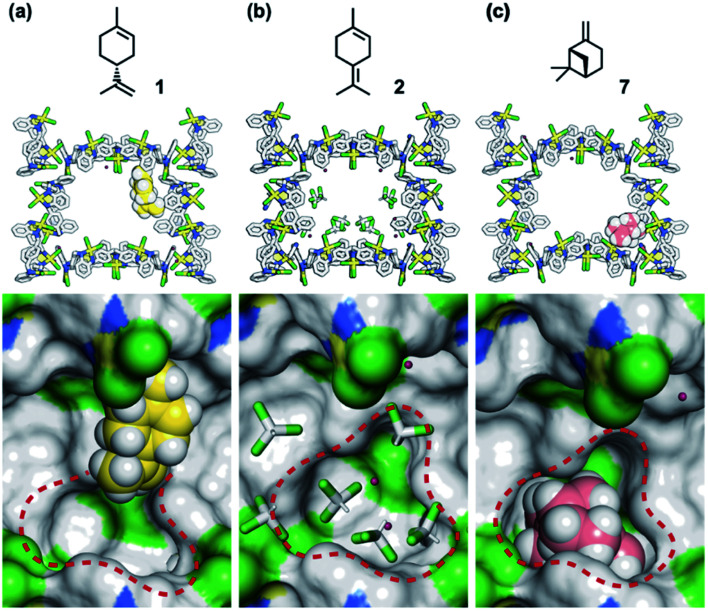
Crystallographic study of MMFs soaked in (a) a CHCl_3_ solution containing 1 (1.0 M), (b) a CHCl_3_ solution containing 2 (1.0 M), and (c) a CH_3_CN solution containing 7 (1.0 M). MMF: stick model or surface model; 1 and 7: space-filling model; water and CHCl_3_: stick model. Red dashed circles indicate the bottom pocket of the MMF. MMF: Pd, yellow; Cl, green; N, blue; C, grey. 1: C, yellow; H, white. 7: C, pink; H, white. Water and CHCl_3_: O, red; H, white; C, grey; Cl, green. Hydrogen atoms attached to the MMF were omitted for clarity. Green and blue surface represents exposed Cl and N–H groups of the MMF, respectively.

The Royal Society of Chemistry apologises for these errors and any consequent inconvenience to authors and readers.

## Supplementary Material
