# Supplementary material for: Highly selective acid-catalyzed olefin isomerization of limonene to terpinolene by kinetic suppression of overreactions in a confined space of porous metal–macrocycle frameworks
Source: Chem Sci. 2022 Jun 3;13(30):8752–8. doi: 10.1039/d2sc01561g (PMC9350587; doi:10.1039/d2sc01561g)
Supplement: SC-013-D2SC01561G-s001 [file SC-013-D2SC01561G-s001.pdf]

Electronic Supplementary Material (ESI) for Chemical Science.
This journal is © The Royal Society of Chemistry 2022

This version of the ESI published 03/08/2022 replaces the previous version published 03/06/2022.
Figure S18 is replaced with a corrected version.

Electronic Supplementary Information (ESI)

Highly selective acid-catalyzed olefin isomerization of limonene to terpinolene by kinetic suppression of the overreactions in a confined space of porous metal-macrocycle framework

Wei He, Shohei Tashiro* and Mitsuhiko Shionoya*

Department of Chemistry, Graduate School of Science, The University of Tokyo, 7-3-1 Hongo, Bunkyo-ku, Tokyo 113-0033, Japan

- **1. Materials and methods** page S2
- **2. Preparation and characterization of 2-NBSA@MMF** page S2
- **3. Isomerization of (+)-limonene using 2-NBSA@MMF and 2-NBSA·H_2O** page S7
- **4. Examination of the inhibitory effects of additives on the over-isomerization** page S14
- **5. Crystal structures of MMF soaked in a solution of terpenes** page S19
- **6. Cyclization of nerol using 2-NBSA@MMF and 2-NBSA·H_2O** page S25
- **7. References** page S29

1. Materials and methods

Metal–macrocycle framework (MMF) and macrocyclic ligand **L** were prepared according to our procedure.[1] MMF crystals were washed with CH_3CN and then used for guest incorporation. 2-Nitrobenzenesulfonic acid monohydrate (2-NBSA·H_2O) was purchased from TCI Co. Ltd. 2-NBSA@MMF was prepared according to a soaking-and-washing procedure which we developed to immobilize *p*-toluenesulfonic acid in MMF.[2] The detailed procedure is described on page S2. The previously reported catalyst, *p*-TsOH@MMF, was also prepared according to our procedure.[2] Commercially available $CHCl_3$ and $CDCl_3$ were passed through basic Al_2O_3 before use to remove a trace amount of HCl or DCl. In this study, only the reaction products that could be directly compared with the standard samples by ^{1}H NMR spectra were attributed. For instance, (+/–)-limonene, terpinolene (**2**), α-terpinene (**3**), γ-terpinene (**4**) and *p*-cymene (**5**) were identified by matching their ^{1}H NMR resonances to commercial samples (**2** was also identified by ^{13}C NMR analysis). Terpene substrates, terpenoid substrates, standards for product assignment, solvents, organic and inorganic reagents were all commercially available, and used without further purification.

NMR spectroscopic measurements were performed using a Bruker AVANCE 500 spectrometer. ^{1}H NMR spectra are calibrated as below: Si(CH_3)$_4$ = 0 ppm in $CDCl_3$, C*H*D_2SOCD_3 = 2.50 ppm in DMSO-d_6. FT-IR spectra were recorded on a JASCO FT/IR-4200 spectrometer using a ZnSe attenuated total reflection (ATR) method. Single-crystal XRD (ScXRD) analyses were performed using an XtaLAB P200 system diffractometer with CuKα-radiation, and the obtained data were analyzed using an Olex2 software package[3] except for refinement, which was performed using a SHELXL-2018/3 program suite.[4] Several restraints were applied to MMF and guest molecules in all the crystal structures. Hydrogen atoms were placed at the calculated positions and refined using a riding model. The occupancies of the guest molecules were refined based on the electron densities using the free variables of the SHELXL-2018/3 program. In assigning guest molecules, all Q peaks above 2.0, except for the ghost peaks around Pd, were assigned by the least-square refinement, and all Q peaks above 1.4 were tested for guest search. The X-ray structures were displayed using the PyMOL program. Electron density maps were generated using the ShelXle program.[5] The non-covalent interactions were analyzed by the reduced density gradient method[6,7] using the NCIPLOT program[8] and visualized by the VMD program.[9]

2. Preparation and characterization of 2-NBSA@MMF

2.1 Procedures for the preparation of 2-NBSA+MMF and 2-NBSA@MMF crystals

MMF crystals (ca. 0.5 mg) were soaked in a CH_3CN solution of 2-NBSA·H_2O (1.0 M, 100 μL, 100 μmol) in a capped micro-tube at 298 K for 24 h to obtain 2-NBSA+MMF crystals. Resulting 2-NBSA+MMF crystals were washed with a small amount of CH_3CN (ca. 100 μL), collected by

filtration, then washed again with $CHCl_3$ (ca. 800 μL). The resulting 2-NBSA@MMF crystals were immediately used for catalytic reactions or characterization. **Caution!** *A CH_3CN solution of 2-NBSA may corrode nitrile gloves, so it is better to wear polyethylene gloves outside the nitrile gloves when handling.*

2.2 Crystal structure of 2-NBSA+MMF and 2-NBSA@MMF

One 2-NBSA+MMF or 2-NBSA@MMF crystal was picked up separately in a micro loop and immediately mixed with paratone oil and single-crystal XRD was measured at 93 K.

Crystal data for 2-NBSA+MMF

Crystal data for $(Pd_3\mathbf{L}Cl_6)_2\cdot(\text{2-NBSA})_{0.37}\cdot(CH_3CN)\cdot(H_2O)_{0.87}$: $C_{88.2}H_{87.5}Cl_{12}N_{13.4}O_{2.7}Pd_6S_{0.4}$, Fw = 2453.43, crystal dimensions 0.14 × 0.15 × 0.22 mm^3, monoclinic, space group $P2_1/c$, a = 19.5405(2), b = 51.93156(5), c = 14.3152(1) Å, β = 90.587(1)°, V = 14525.8(2) Å^3, Z = 4, ρ_{calcd} = 1.122 g cm^{-3}, μ = 8.23 mm^{-1}, T = 93 K, λ(CuKα) = 1.54187 Å, $2\theta_{max}$ = 148.6°, 168241/28625 reflections collected/unique (R_{int} = 0.0517), R_1 = 0.1209 ($I > 2\sigma(I)$), wR_2 = 0.3983 (for all data), GOF = 1.665, largest diff. peak and hole 4.110/–1.700 eÅ^{-3}. CCDC deposit number 2133387. Several restraints were applied to the MMF and the guest molecules to prevent the structure from collapsing during the least-squares refinement.

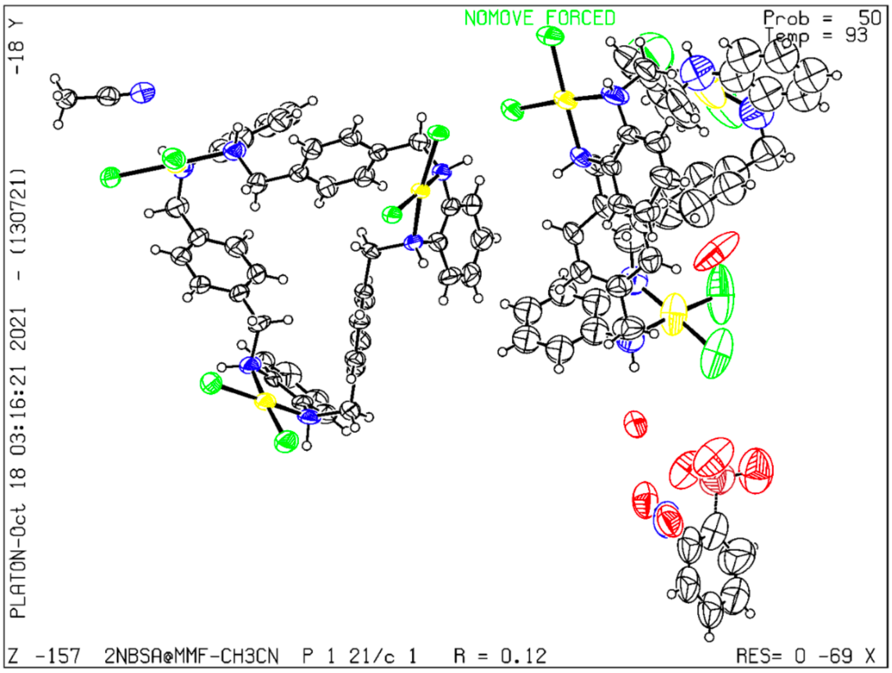

Fig. S1 ORTEP drawing of 2-NBSA+MMF at the 50% probability level. Color: C black, N blue, O red, Cl green, S deep red and Pd yellow.

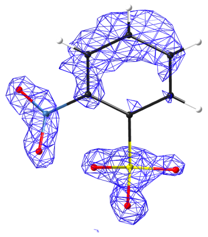

Fig. S2 Electron density maps of 2-NBSA in the crystal structure of 2-NBSA+MMF (contour level: 1.00 e^{-}/Å^{3}).

<u>Crystal data for 2-NBSA@MMF</u>

Crystal data for $(Pd_3\mathbf{L}Cl_6)_2\cdot(CH_3CN)\cdot(H_2O)$: $C_{86}H_{87}Cl_{12}N_{13}O_2Pd_6$, Fw = 2382.48, crystal dimensions 0.10 × 0. 10 × 0.21 mm^3, monoclinic, space group $P2_1/c$, a = 19.6017(1), b = 52.3627(4), c = 14.3397(1) Å, β = 90.600(1)°, V = 14717.43(17) Å^3, Z = 4, ρ_{calcd} = 1.075 g cm^{-3}, μ = 8.06 mm^{-1}, T = 93 K, λ(CuKα) = 1.54187 Å, $2\theta_{max}$ = 147.1°, 173429/28355 reflections collected/unique (R_{int} = 0.0533), R_1 = 0.1080 ($I > 2\sigma(I)$), wR_2 = 0.3596 (for all data), GOF = 1.546, largest diff. peak and hole 4.228/–2.216 eÅ^{-3}. CCDC deposit number 2133388. Several restraints were applied to the MMF and the guest molecules to prevent the structure from collapsing during the least-squares refinement.

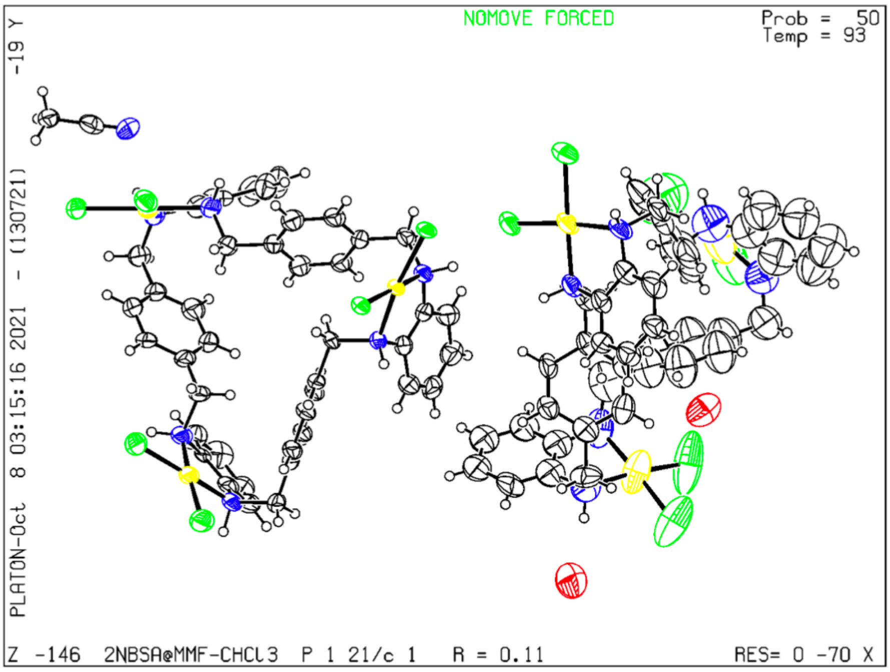

Fig. S3 ORTEP drawing of 2-NBSA@MMF at the 50% probability level. Color: C black, N blue, O red, Cl green and Pd yellow.

2.3 FT-IR measurement of 2-NBSA@MMF

To confirm the presence of 2-NBSA in the 2-NBSA@MMF crystals, the as-synthesized 2-NBSA@MMF crystals were air-dried on a filter paper for 15 sec, and then examined by ATR-IR measurement at room temperature.

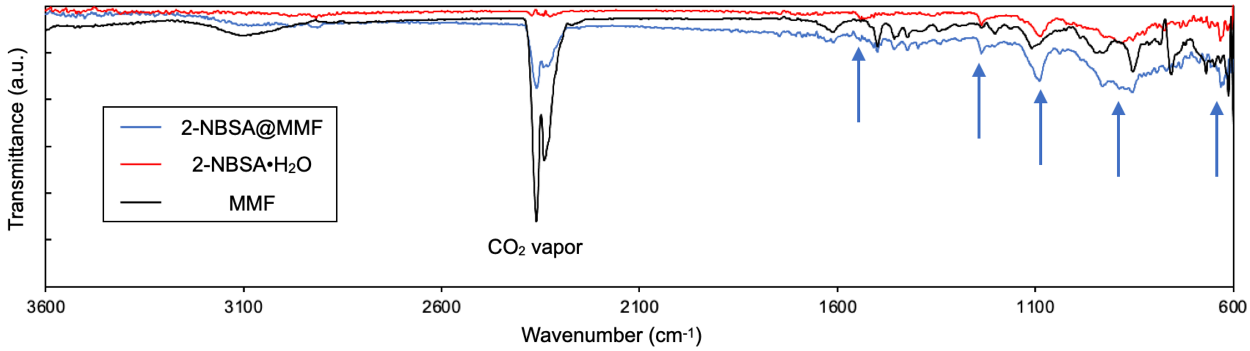

Fig. S4 FT-IR spectra (ATR, rt) of 2-NBSA@MMF crystals, 2-NBSA·H_2O, and as-synthesized MMF crystals. The peaks indicated by blue arrows indicate the presence of 2-NBSA in the 2-NBSA@MMF crystals.

2.4 Digestion NMR experiments of 2-NBSA@MMF

To estimate the amount of 2-NBSA in the unit space of 2-NBSA@MMF crystals, the as-synthesized 2-NBSA@MMF crystals were air-dried on a filter paper for 45 sec, then digested DMSO-d_6/DCl-D_2O ([DCl] = 0.17 M), and ^1H NMR was measured at 300 K. As a result, the amounts of 2-NBSA and $CHCl_3$ in the unit space of MMF were estimated to be 1.1 and 3.4 molecules, respectively, based on the integral ratio. For 2-NBSA+MMF, the amount of 2-NBSA in the unit space of the MMF was estimated to be 2.8 molecules from the integral ratio. Here, the "unit space" of MMF is defined as a half of the unit cell, and thus corresponds to one unit of a nano-channel of MMF.

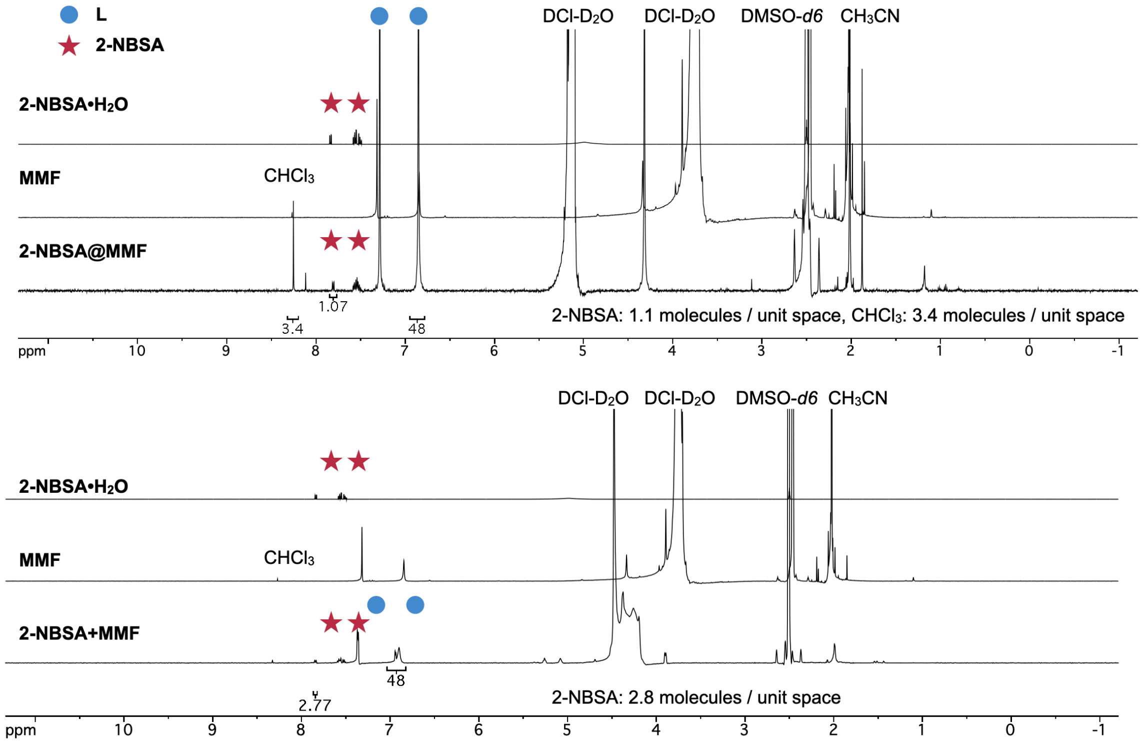

Fig. S5 ^{1}H NMR spectra (500 MHz, 300 K) of 2-NBSA·H_2O, MMF, and 2-NBSA@MMR (top) or 2-NBSA+MMF (bottom) after dissolution. The amounts of 2-NBSA and $CHCl_3$ in the unit space of MMF were estimated and labeled (bottom right). Solvents: DMSO-d_6 for 2-NBSA·H_2O; DMSO-d_6/DCl ([DCl] = 0.32 M) for MMF; DMSO-d_6/DCl ([DCl] = 0.17 M) for 2-NBSA@MMF; DMSO-d_6/DCl ([DCl] = 0.22 M) for 2-NBSA+MMF.

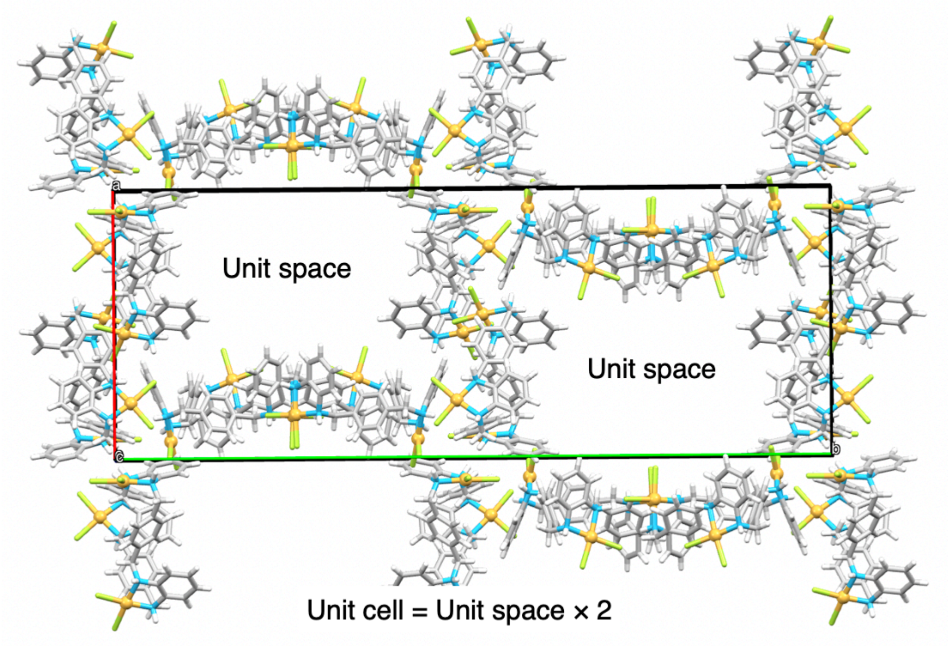

Fig. S6 The definition of the "unit space" of MMF.

2.5 Catalyst quantity of 2-NBSA@MMF

The chemical composition of 2-NBSA@MMF except for water was estimated to be $(Pd_3\mathbf{L}Cl_6)_4\cdot(\text{2-NBSA})_{1.1}\cdot(CHCl_3)_{3.4}\cdot(H_2O)_n$ from the integral ratios of the ^{1}H NMR spectrum (Fig. S5). The number of water molecules included in 2-NBSA@MMF was tentatively estimated to be n = 20 (Mw = 5640.8), since previous elemental analysis suggested that the as-synthesized MMF crystals ($(Pd_3\mathbf{L}Cl_6)_4\cdot(CH_3CN)_2\cdot(H_2O)_{36}$) contained 36 water molecules in its unit space.[1] Therefore, the number n and the molecular weight Mw of 2-NBSA @MMF should be within the ranges of $0 \leqq n \leqq 36$ and $5280.5 \leqq Mw \leqq 5929.1$, respectively. Therefore, the catalyst quantity of 2-NBSA@MMF used in this study (n = 20, Mw = 5640.8) falls within the error range of 6% with respect to water content.

2.6 Acid-leaching test of 2-NBSA@MMF in $CDCl_3$

To examine whether 2-NBSA elutes from 2-NBSA@MMF into $CDCl_3$ solutions, the as-synthesized 2-NBSA@MMF crystals (ca. 0.5 mg) were soaked in $CDCl_3$ (ca. 1.0 mL) at 298 K for 120 h, and analyzed by ^{1}H NMR at 298 K. As the result, acid-leaching was not observed after soaking 2-NBSA@MMF in $CDCl_3$ at 298 K for 120 h.

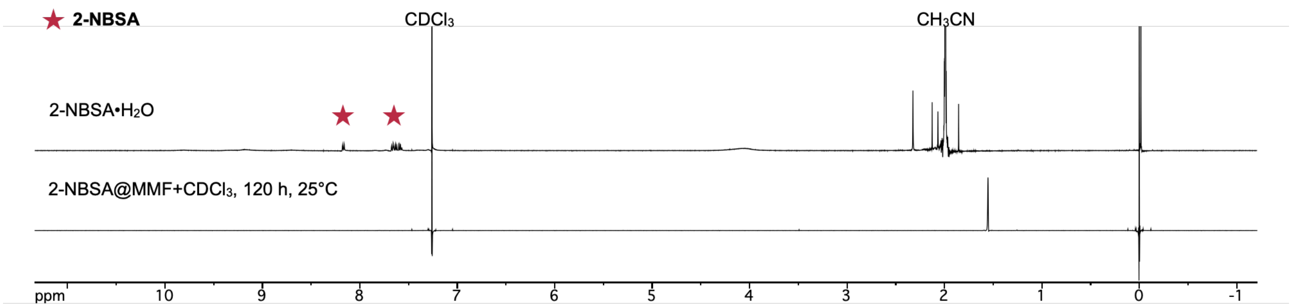

Fig. S7 ^{1}H NMR spectra (500 MHz, $CDCl_3$, 300 K) of a $CDCl_3$ solution containing dissolved 2-NBSA·H_2O or a solution heterogeneously containing 2-NBSA@MMF crystals.

2.7 Catalytic activity tests of 2-NBSA@MMF and *p*-TsOH@MMF

In the heterogeneous reactions, a $CDCl_3$ solution of acetaldehyde diethyl acetal (100 mM, 1.0 mM, 100 μmol) was mixed with 2-NBSA@MMF or *p*-TsOH@MMF (acid in MMF, 1 mol%) in an NMR tube. For the homogeneous reactions, a $CDCl_3$ solution of acetaldehyde diethyl acetal (100 mM, 1.0 mM, 99 μmol) containing 2-NBSA·H_2O or *p*-TsOH·H_2O (2 mol%) was prepared in an NMR tube. The mixtures were shaken on a shaker at 298 K and monitored by ^{1}H NMR spectroscopy (500 MHz $CDCl_3$, 300 K).

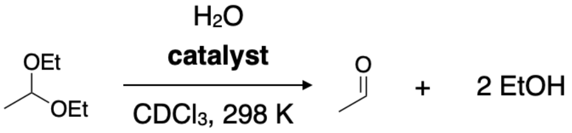

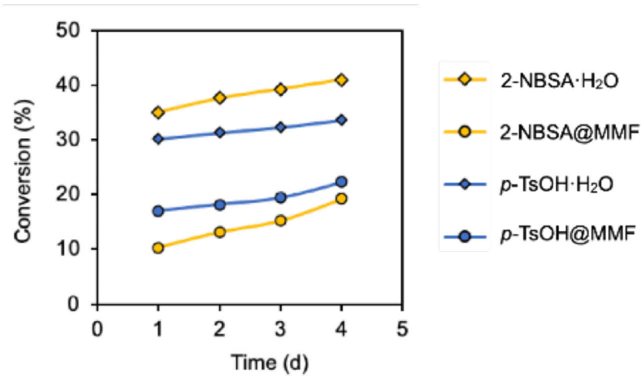

Fig. S8 Catalytic hydrolysis reaction of acetaldehyde diethyl acetal with 2-NBSA@MMF, *p*-TsOH@MMF, 2-NBSA·H_2O, or *p*-TsOH·H_2O in $CDCl_3$ at 298 K.

3. Isomerization of (+)-limonene using 2-NBSA@MMF and 2-NBSA·H_2O

3.1 Procedure for the isomerization of (+)-limonene using 2-NBSA@MMF

Crystals of 2-NBSA@MMF (ca. 0.5 mg, 0.1 μmol of 2-NBSA in MMF, 1 mol%) immediately after preparation and a $CDCl_3$ solution of (+)-limonene (**1**) (10 mM, 1.0 mL, 10 μmol) were placed in an NMR tube. The heterogeneous mixture was shaken on a shaker at 298 K and the time course was monitored by ^{1}H NMR measurements (500 MHz, $CDCl_3$, 300 K). The conversion ratios of (+)-

limonene (**1**) and yields of main products were evaluated using 1,1,2,2-tetrachloroethane (9 mM, 1.0 mL, 9 μmol) as internal standard. When (+)-limonene (**1**) was treated with 2-NBSA@MMF, 45% of (+)-limonene (**1**) was consumed at 298 K after 51 h, and the formation of terpinolene (**2**) (41% yield, 91% selectivity), α-terpinene (**3**) (0.6%), γ-terpinene (**4**) (1.7%), and *p*-cymene (**5**) (0.6%) was confirmed. When the reaction was further extended to 100 h at 298 K, 67% of (+)-limonene (**1**) was consumed and the formation of terpinolene (**2**) (50% yield, 75% selectivity), α-terpinene (**3**) (3%), γ-terpinene (**4**) (4%) and *p*-cymene (**5**) (3%) was confirmed. Next, to further confirm the formation of terpinolene (**2**), a similar reaction mixture was prepared separately under the same conditions and analyzed by ^{13}C NMR spectroscopy (126 MHz, $CDCl_3$, 300 K) after 57 h. As a result, **2** was identified by matching the ^{13}C NMR resonances with a commercial sample.

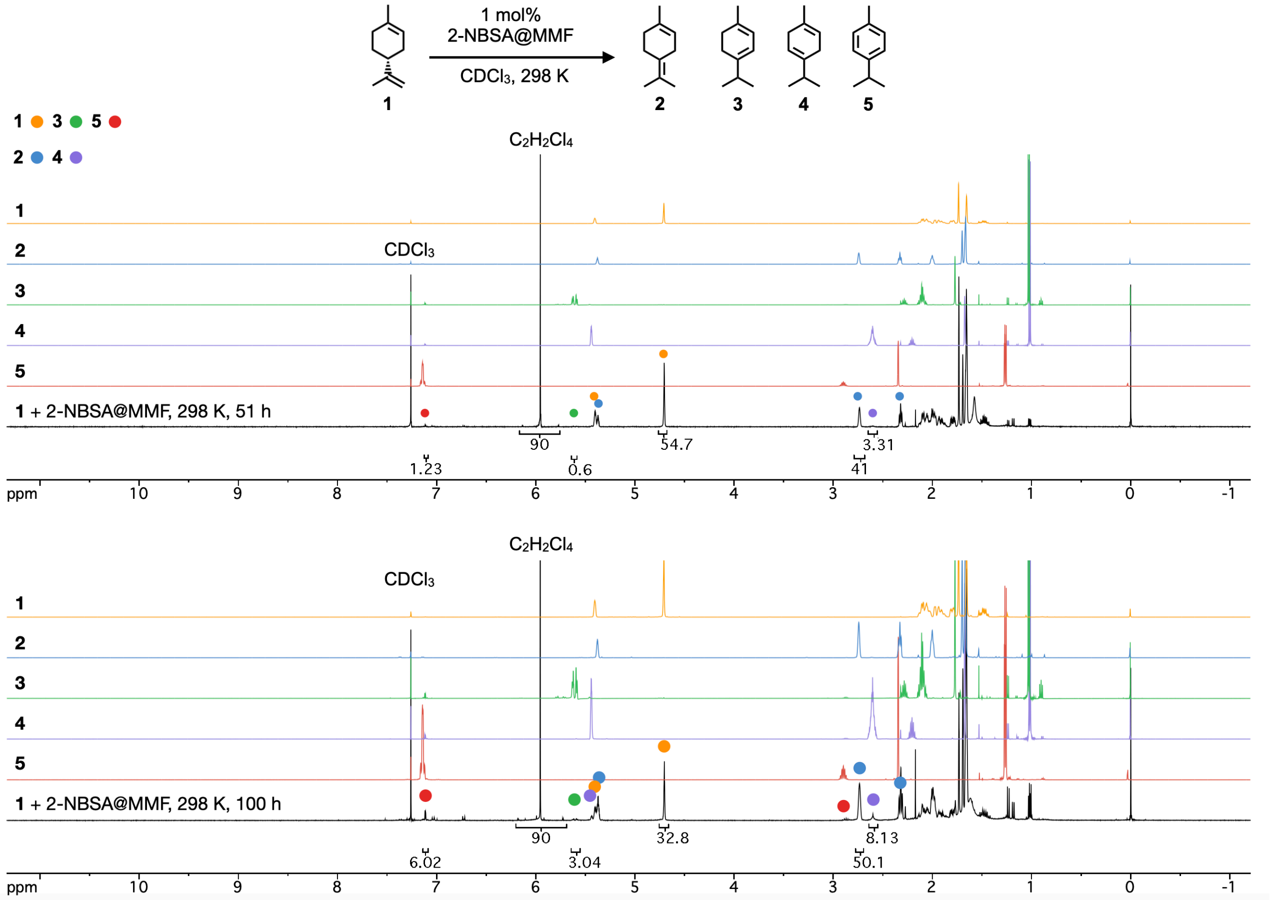

Fig. S9 ^{1}H NMR spectra (500 MHz, $CDCl_3$, 300 K) of **1**–**5** and the reaction mixtures of (+)-limonene (**1**) and 2-NBSA@MMF in $CDCl_3$ at 298 K after 51 h (top) and 100 h (bottom).

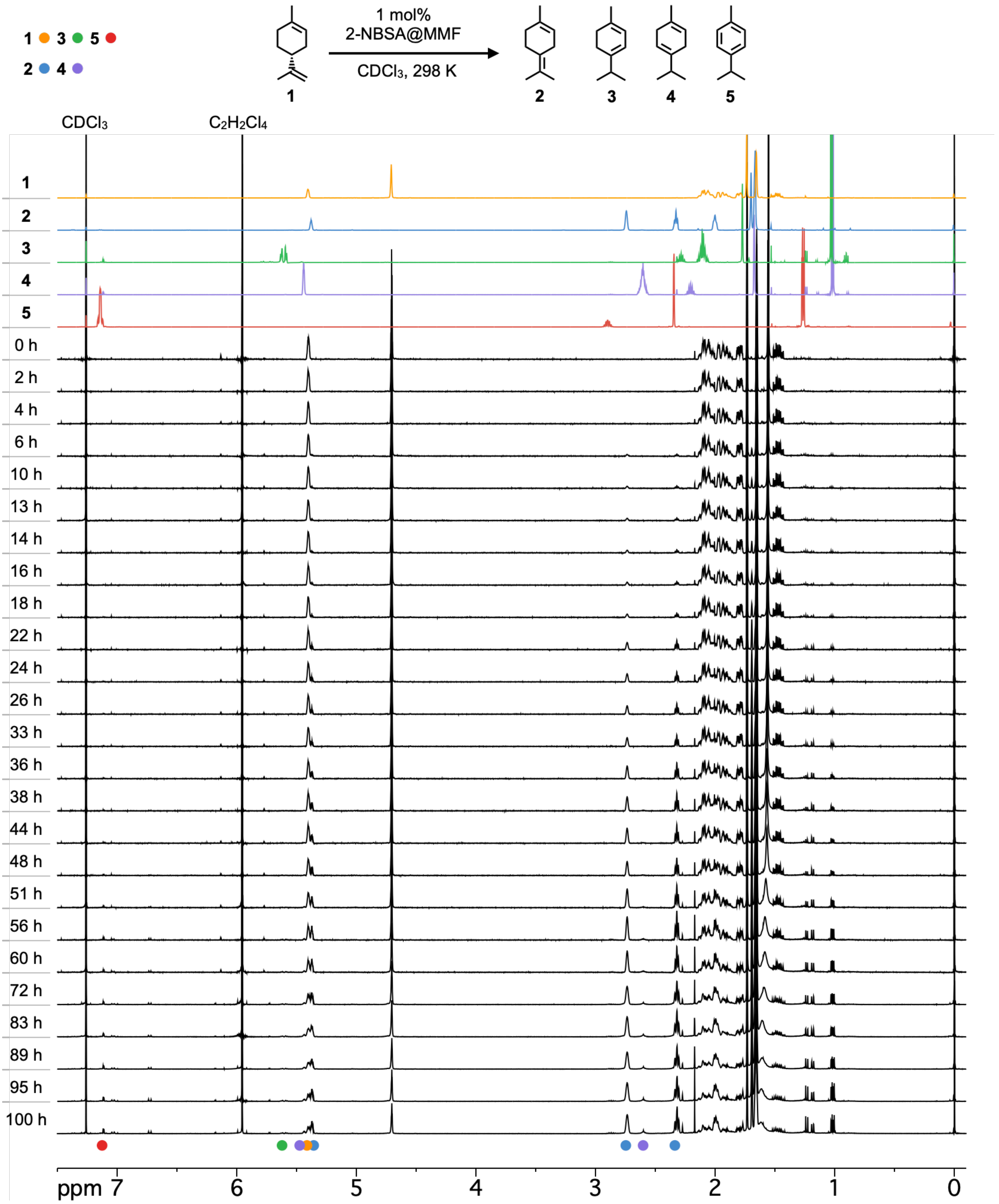

Fig. S10 ^{1}H NMR spectra (500 MHz, $CDCl_3$, 300 K) of **1**–**5** and the time-course analysis of the reaction mixture of (+)-limonene (**1**) and 2-NBSA@MMF in $CDCl_3$ at 298 K.

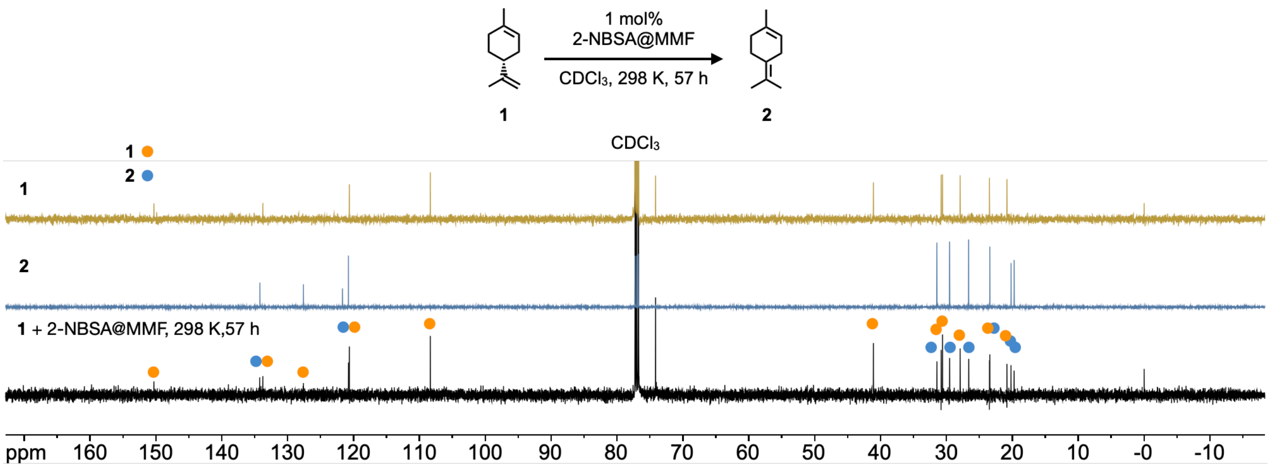

Fig. S11 ^{13}C NMR spectra (126 MHz, $CDCl_3$, 300 K) of **1**, **2**, and the reaction mixture of (+)-limonene (**1**) and 2-NBSA@MMF in $CDCl_3$ at 298 K after 57 h.

To investigate the effect of catalyst amount on this reaction, the catalyst was increased to 3 mol% as follows. First, crystals of 2-NBSA@MMF (ca. 1.5 mg, 0.3 µmol of 2-NBSA in MMF, 3 mol%) immediately after preparation and a $CDCl_3$ solution of (+)-limonene (**1**) (10 mM, 1.0 mL, 10 µmol) were placed in an NMR tube. The heterogeneous mixture was shaken on a shaker at 298 K and the time course was monitored by ^{1}H NMR measurements (500 MHz, $CDCl_3$, 300 K). The conversion ratios of (+)-limonene (**1**) and the yields of the main products were evaluated using 1,1,2,2-tetrachloroethane (9 mM, 1.0 mL, 9 µmol) as internal standard. When (+)-limonene (**1**) was treated with 2-NBSA@MMF, 85% of (+)-limonene (**1**) was consumed at 298 K after 100 h, and the formation of terpinolene (**2**) (41% yield, 48% selectivity), α-terpinene (**3**) (2%), γ-terpinene (**4**) (6%), and *p*-cymene (**5**) (10%) was confirmed.

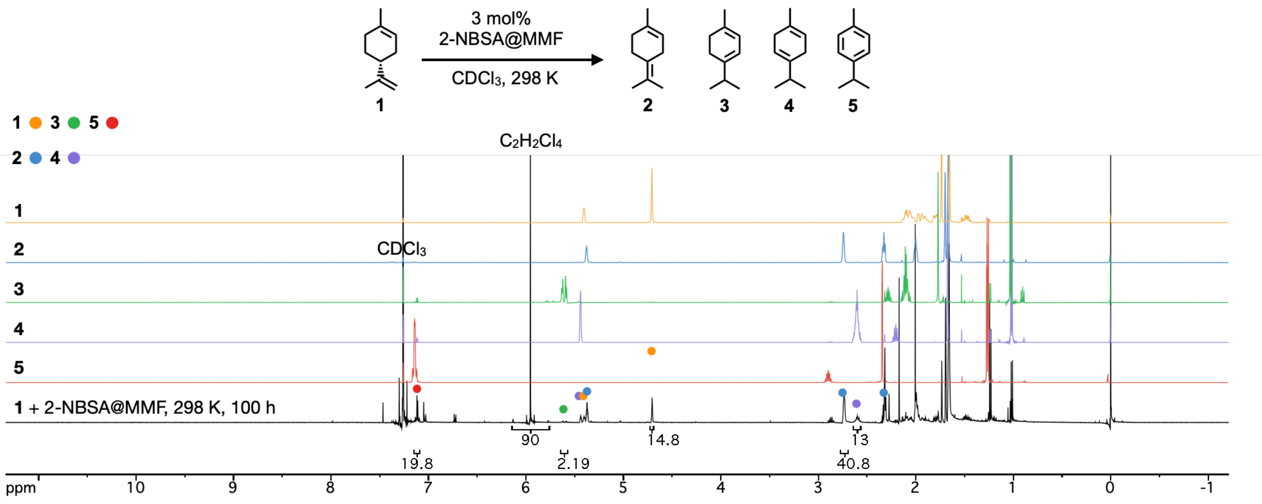

Fig. S12 ^{1}H NMR spectra (500 MHz, $CDCl_3$, 300 K) of **1–5** and the reaction mixtures of (+)-limonene (**1**) and 2-NBSA@MMF in $CDCl_3$ at 298 K after 100 h.

3.2 Confirmation of heterogeneous properties of the 2-NBSA@MMF catalyst

Crystals of 2-NBSA@MMF (ca. 0.5 mg, 0.1 µmol of 2-NBSA in MMF, 1 mol%) immediately after preparation and a $CDCl_3$ solution of (+)-limonene (10 mM, 1.0 mL, 10 µmol) were placed in an NMR tube. 1,1,2,2-Tetrachloroethane (7.5 mM, 1.0 mL, 7.5 µmol) was used as internal standard to evaluate the conversion ratios of (+)-limonene (**1**) and the yields of the main products. This heterogeneous mixture was shaken on a shaker at 298 K for 36 h, and ^{1}H NMR measurements revealed that 30% of **1** was converted to **2**–**5**.

Then, half of the supernatant was removed with a syringe and filtrated through a membrane filter unit. The supernatant and the rest of the suspension were placed separately under the same conditions. After 17 h, ^{1}H NMR measurement showed that the conversion rate of the supernatant liquid had hardly increased (31%), while the conversion rate of the suspension had reached 46%.

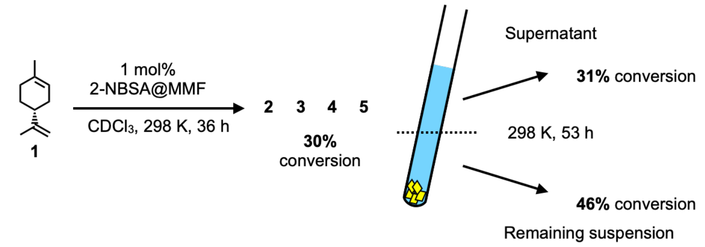

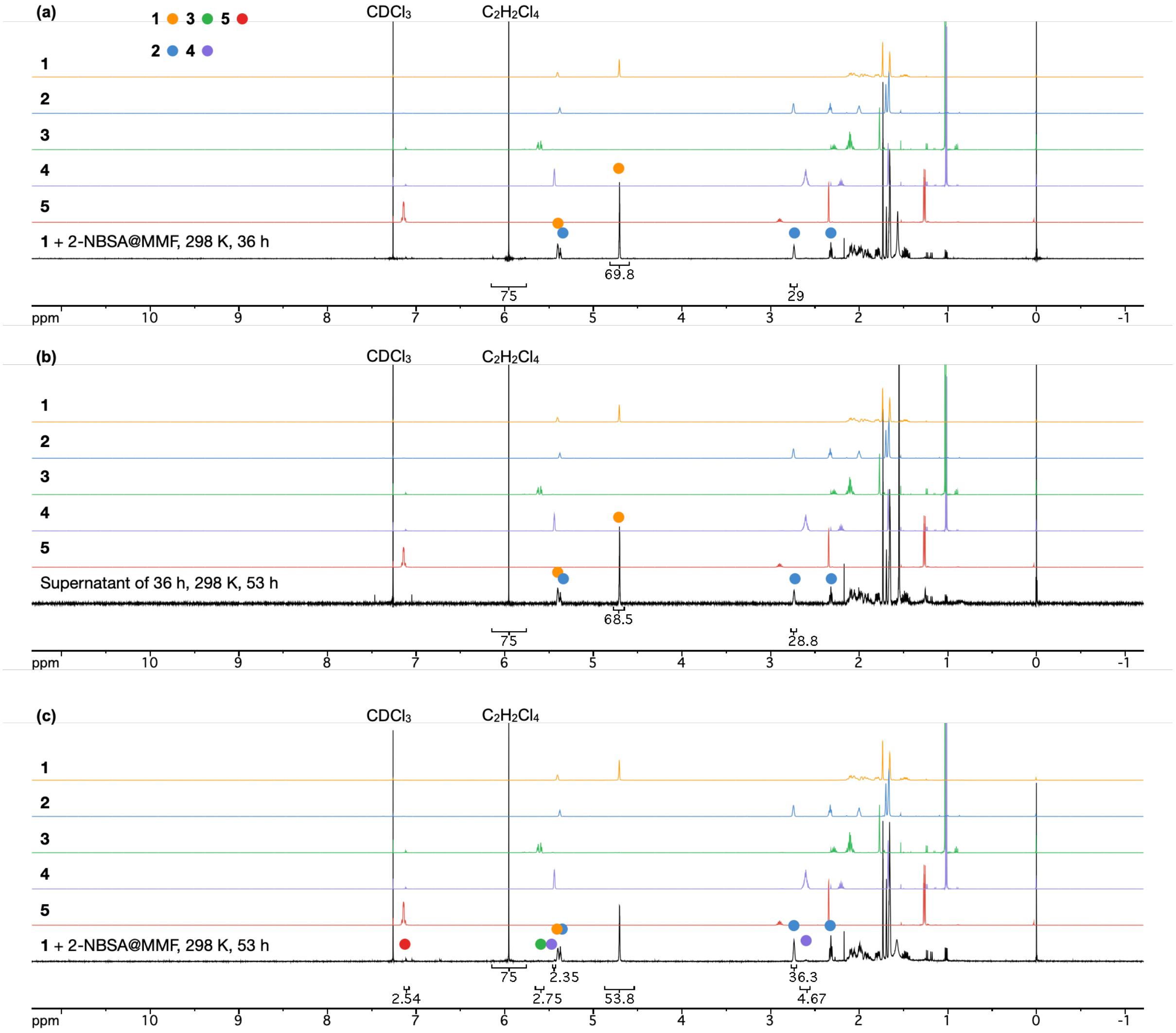

Fig. S13 ^{1}H NMR spectra (500 MHz, $CDCl_3$, 300 K) of **1**–**5**, and (a) the reaction mixture of **1** and 2-NBSA after 36 h; (b) the supernatant of the 36 h-reaction after 53 h; (c) the remaining suspension of the 36 h-reaction after 53 h.

3.3 Procedure for the isomerization of (+)-limonene using 2-NBSA·H_2O

A $CDCl_3$ solution of 2-NBSA·H_2O (1 mM, 0.1 mL, 0.1 μmol, 1 mol%) and a $CDCl_3$ solution of (+)-limonene (**1**) (11 mM, 0.9 mL, 9.9 μmol) were set in an NMR tube. This mixture was shaken on a shaker at 298 K and the time course was monitored by ^{1}H NMR measurements (500 MHz, $CDCl_3$, 300 K). 1,1,2,2-Tetrachloroethane (4.5 mM, 1.0 mL, 4.5 μmol) was used as an internal standard to evaluate the conversion ratio of (+)-limonene and the yield of main product. Treatment of (+)-limonene (**1**) with 2-NBSA·H_2O at 298 K for 12 h resulted in 80% conversion of (+)-limonene (**1**) to terpinolene (**2**) (50% yield, 63% selectivity), α-terpinene (**3**) (2.2%), γ-terpinene (**4**) (1.8%) and *p*-cymene (**5**) (7.5%). After extending the reaction to 100 h at 298 K, 98% of (+)-limonene (**1**) was converted to terpinolene (**2**) (9.8% yield, 10% selectivity), α-terpinene (**3**) (5.8%), γ-terpinene (**4**) (8.0%) and *p*-cymene (**5**) (30%).

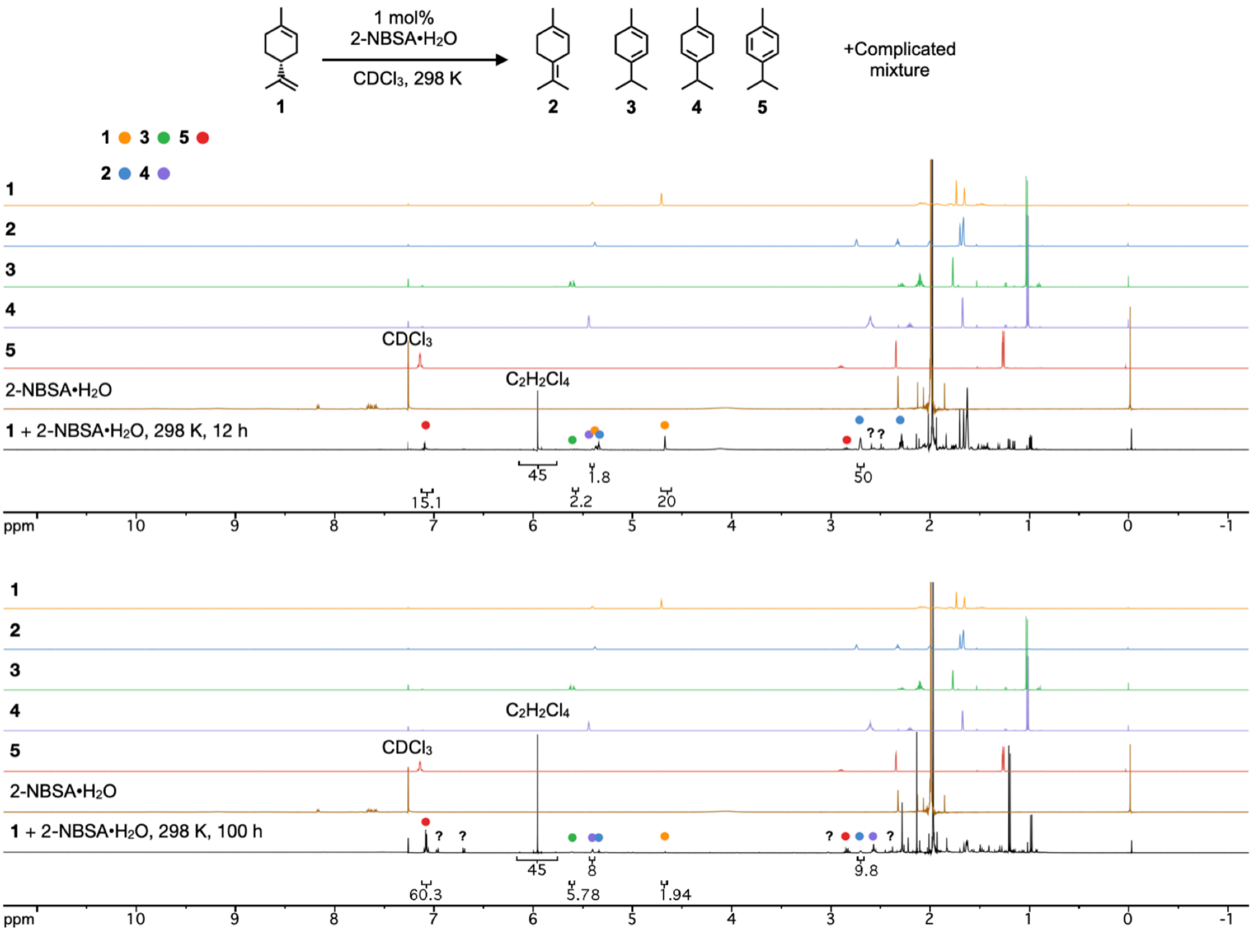

Fig. S14 ^{1}H NMR spectra (500 MHz, $CDCl_3$, 300 K) of **1**–**5** and the reaction mixture of (+)-limonene (**1**) and 2-NBSA·H_2O in $CDCl_3$ at 298 K after 12 h (top) and 100 h (bottom).

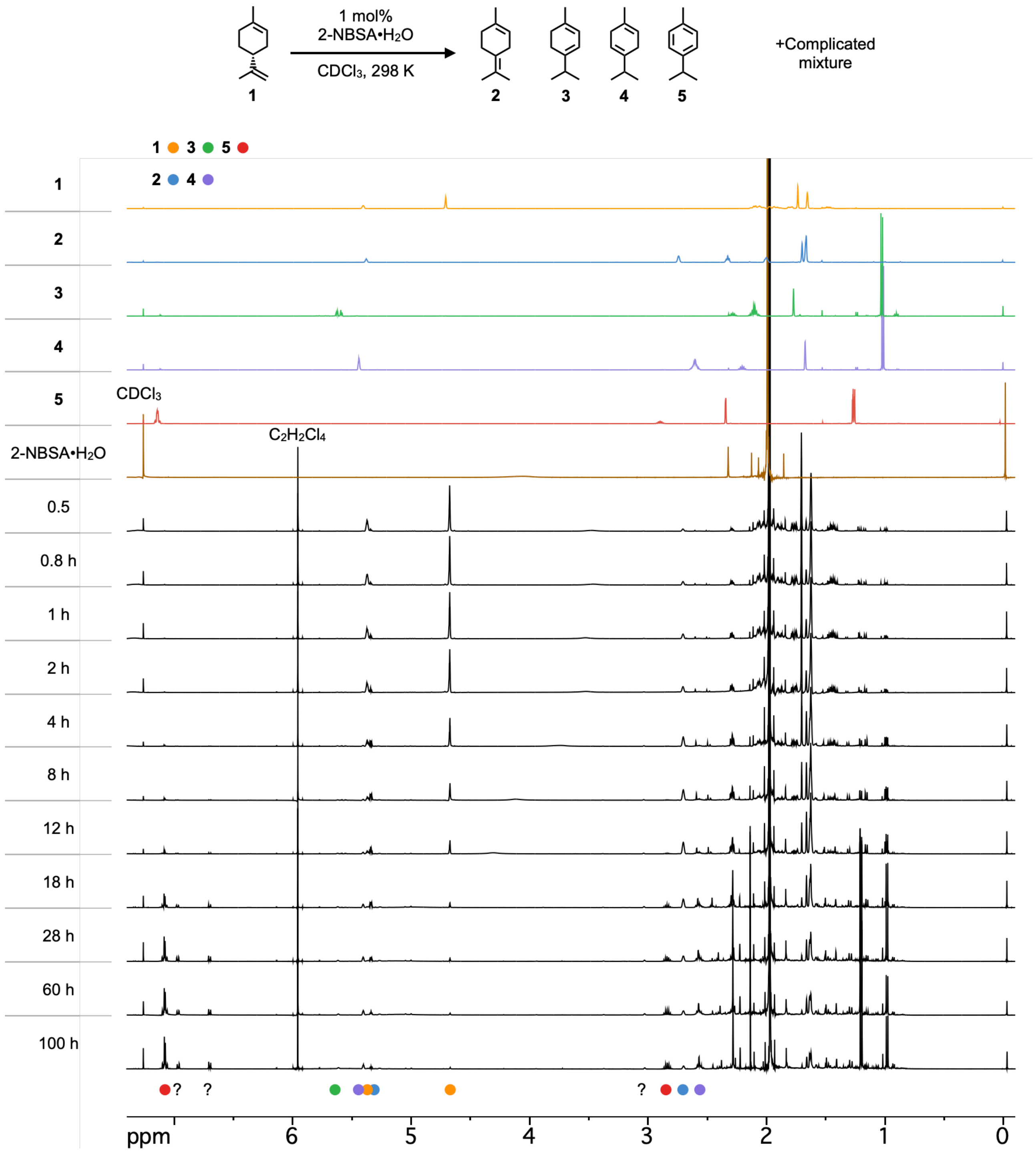

Fig. S15 ^{1}H NMR spectra (500 MHz, $CDCl_3$, 300 K) of **1**–**5**, 2-NBSA·H_2O, and the time-course analysis of the reaction mixture of (+)-limonene (**1**) and 2-NBSA·H_2O in $CDCl_3$ at 298 K.

3.4 Reproducibility test of the catalytic reactions

Catalytic conversion of (+)-limonene (**1**) with 2-NBSA@MMF or 2-NBSA·H_2O was repeated three times under the same condition to verify the reproducibility. The standard errors in Fig. 4d were estimated from these results.

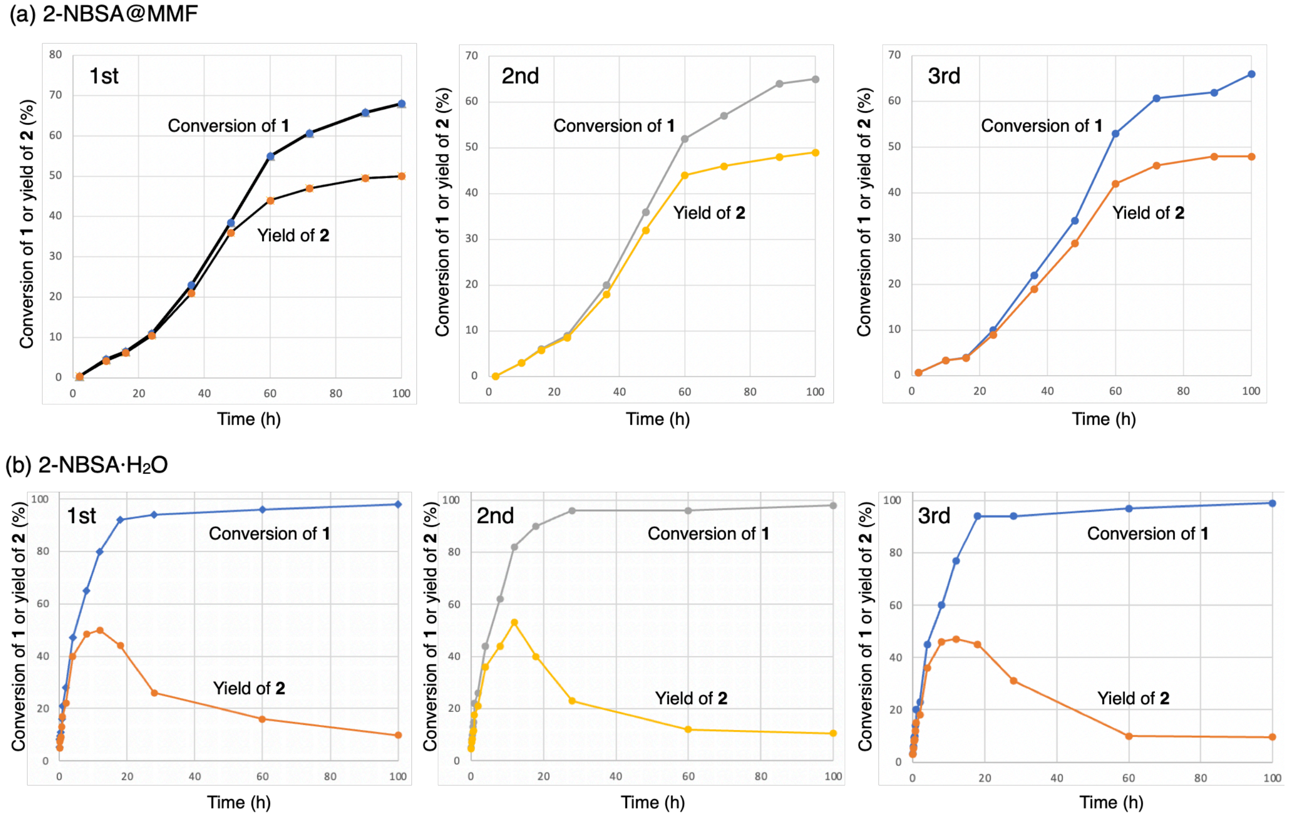

Fig. S16 Time-course analysis of the isomerization reaction of **1** with 1 mol% of (a) 2-NBSA@MMF or (b) 2-NBSA·H_2O in $CDCl_3$ at 298 K.

4. Examination of the inhibitory effects of additives on the over-isomerization

4.1 Inhibitory effects of (+)-limonene on the over-isomerization of terpinolene using 2-NBSA@MMF

To confirm the over-isomerization of terpinolene (**2**) using 2-NBSA@MMF, crystals of 2-NBSA@MMF (ca. 0.5 mg, 0.1 µmol of 2-NBSA in MMF, 1 mol%) immediately after preparation and a $CDCl_3$ solution of terpinolene (**2**) (10 mM, 1.0 mL, 10 µmol) were set in an NMR tube. This heterogeneous mixture was shaken on a shaker at 298 K and analyzed by ^{1}H NMR measurements (500 MHz, $CDCl_3$, 300 K) after 102 h. The conversion ratios of terpinolene (**2**) was evaluated from the internal standard of 1,1,2,2-tetrachloroethane (9 mM, 1.0 mL, 9 µmol). Terpinolene (**2**) was treated with 2-NBSA@MMF, 54% of terpinolene (**2**) was consumed and the formation of α-terpinene (**3**) (5%), γ-terpinene (**4**) (11%), *p*-cymene (**5**) (14%) and limonene (8%) at 298 K for 102 h.

Then, to examine the inhibitory effects of (+)-limonene (**1**) on over-isomerization, 30 mol%, 100 mol%, or 300 mol% of (+)-limonene (**1**) (3 µmol, 10 µmol, or 30 µmol, respectively, based on terpinolene) were added to the reaction mixture before shaking at 298 K for 102 h. The conversion of terpinolene (**2**) to α-terpinene (**3**), γ-terpinene (**4**) and *p*-cymene (**5**) decreased to 47%, 37% and 16%, respectively.

Tab. S1 Inhibitory effects of (+)-limonene (**1**) on over-isomerization of terpinolene (**2**).

Additives	Concentration (mM)				Conversion (**2**)[a]
	$[\mathbf{1}]_{0h}$	$[\mathbf{2}]_{0h}$	$[\mathbf{1}]_{102h}$	$[\mathbf{2}]_{102h}$	
30 mol% **1**	3	10	1.8	6.5	47%
100 mol% **1**	10	10	5.0	11.3	37%
300 mol% **1**	30	10	12.1	26.3	16%

[a] Considering that additive **1** selectively converted to **2**, the conversion rate of **2** was calculated as follows, where ($[\mathbf{1}]_{0h} - [\mathbf{1}]_{102h}$) represents the change in concentration of **1** after 102 h.

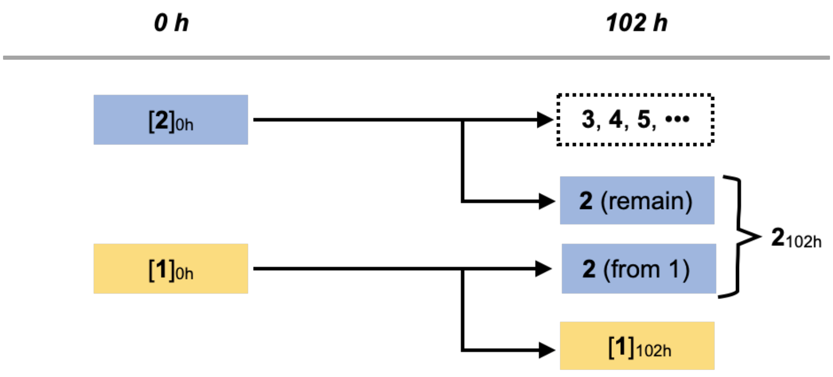

$$\text{Conversion } (\mathbf{2}) = \frac{[\mathbf{2}]_{0h} - [\mathbf{2}]_{remain}}{[\mathbf{2}]_{0h}} = \frac{[\mathbf{2}]_{0h} - \{[\mathbf{2}]_{102h} - ([\mathbf{1}]_{0h} - [\mathbf{1}]_{102h})\}}{[\mathbf{2}]_{0h}}$$

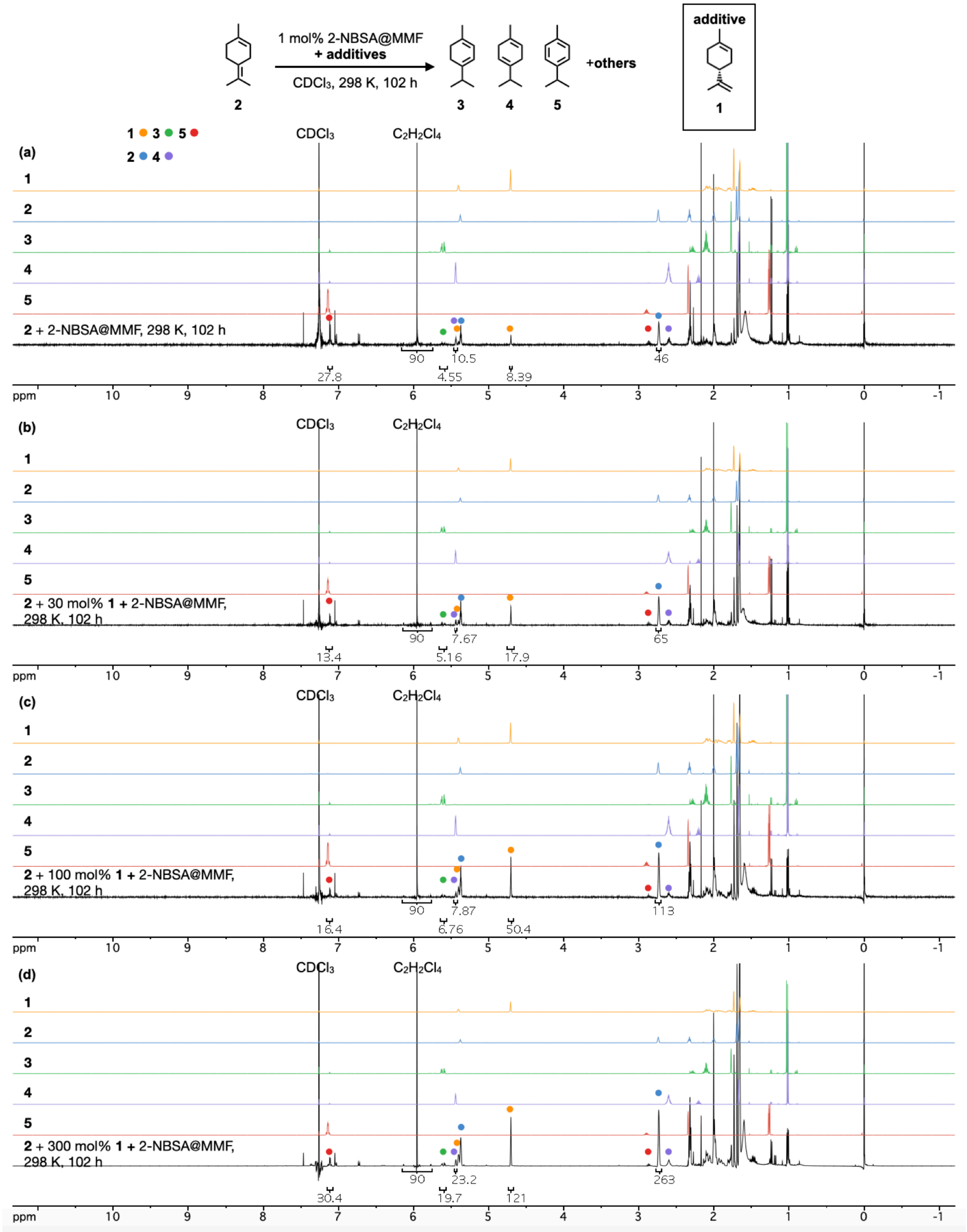

Fig. S17 ^{1}H NMR spectra (500 MHz, $CDCl_3$, 300 K) of **1**–**5**, and (a) the reaction mixture of **2** and 2-NBSA@MMF; (b) the reaction mixture of **2** + 30 mol% of **1** and 2-NBSA@MMF; (c) the reaction mixture of **2** + 100 mol% of **1** and 2-NBSA@MMF; (d) the reaction mixture of **2** + 300 mol% of **1** and 2-NBSA@MMF in $CDCl_3$ at 298 K after 102 h.

4.2 Inhibitory effects of (–)-α-pinene, (–)-β-pinene, benzene, and 1,2-dibromobenzene on the over-isomerization of terpinolene using 2-NBSA@MMF

Crystals of 2-NBSA@MMF (ca. 0.5 mg, 0.1 μmol of 2-NBSA in MMF, 1 mol%) immediately after preparation, a $CDCl_3$ solution of terpinolene (**2**) (10 mM, 1.0 mL, 10 μmol), and additives such as (–)-α-pinene (**6**) (15 μmol, 150 mol% relative to **2**), (–)-β-pinene (**7**) (15 μmol, 150 mol% relative to **2**), benzene (**8**) (19 μmol, 190 mol% relative to **2**) or 1,2-dibromobenzene (**9**) (19 μmol, 190 mol% relative to **2**) were set in an NMR tube. These heterogeneous mixtures were shaken on a shaker at 298 K and analyzed by ^{1}H NMR measurements (500 MHz, $CDCl_3$, 300 K) after 102 h. The conversion ratios of terpinolene (**2**) were evaluated from the internal standard of 1,1,2,2-tetrachloroethane (12 mM, 1.0 mL, 12 μmol). As a result, the conversion of terpinolene (**2**) to α-terpinene (**3**), γ-terpinene (**4**) and *p*-cymene (**5**) decreased to 0%, 0%, 87% and 55%, respectively, at 298 K after 102 h.

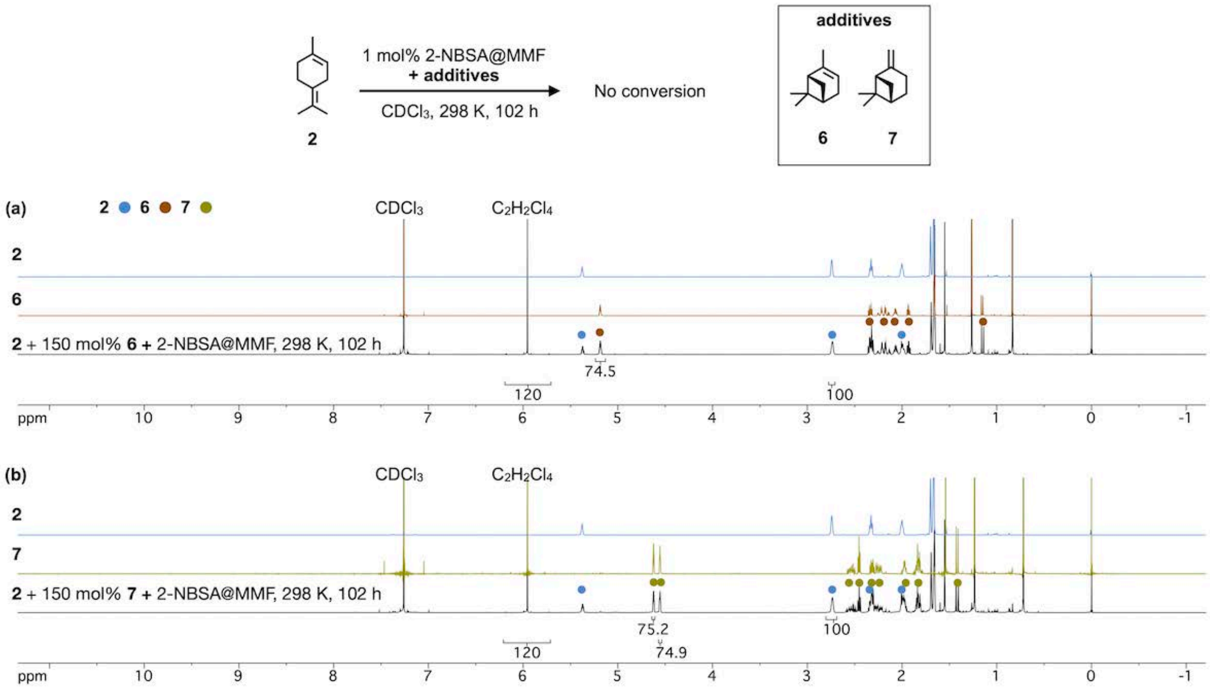

Fig. S18 ^{1}H NMR spectra (500 MHz, $CDCl_3$, 300 K) of (a) **2**, **6**, and the reaction mixture of **2** + 150 mol% of **6** and 2-NBSA@MMF in $CDCl_3$ at 298 K after 102 h; (b) **2**, **7** and the reaction mixture of **2** + 150 mol% of **7** and 2-NBSA@MMF in $CDCl_3$ at 298 K after 102 h.

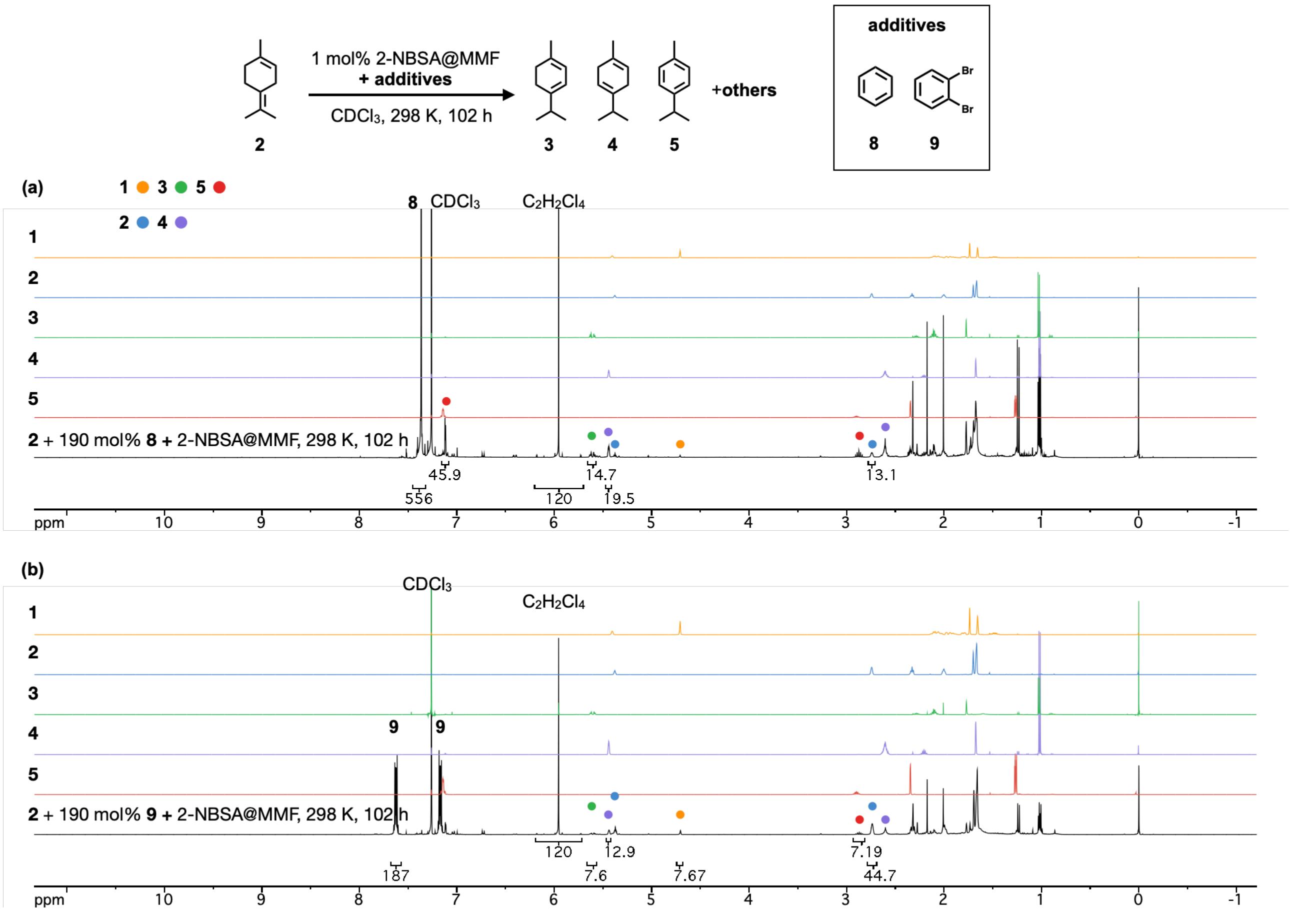

Fig. S19 ^{1}H NMR spectra (500 MHz, $CDCl_3$, 300 K) of **1**–**5**, and (a) the reaction mixture of **2** + 190 mol% of **8** in $CDCl_3$ at 298 K after 102 h; (b) the reaction mixture of **2** + 190 mol% of **9** and 2-NBSA@MMF in $CDCl_3$ at 298 K after 102 h.

4.3 Isomerization of terpinolene using 2-NBSA·H_2O

A $CDCl_3$ solution of 2-NBSA·H_2O (1 mM, 0.1 mL, 0.1 μmol, 1 mol%) and a $CDCl_3$ solution of terpinolene (**2**) (11 mM, 0.9 mL, 9.9 μmol) were set in an NMR tube. This mixture was shaken on a shaker at 298 K and the time course of the reaction was monitored by ^{1}H NMR measurements (500 MHz, $CDCl_3$, 300 K). Using 1,1,2,2-tetrachloroethane (4.5 mM, 1.0 mL, 4.5 μmol) as an internal standard, the conversion ratios of terpinolene (**2**) and the yields of main products were evaluated. Treatment of terpinolene (**2**) with 2-NBSA·H_2O at 298 K for 102 h resulted in consumption of 68% of **2** to α-terpinene (**3**) (4%), γ-terpinene (**4**) (12%), *p*-cymene (**5**) (20%) and limonene (7%).

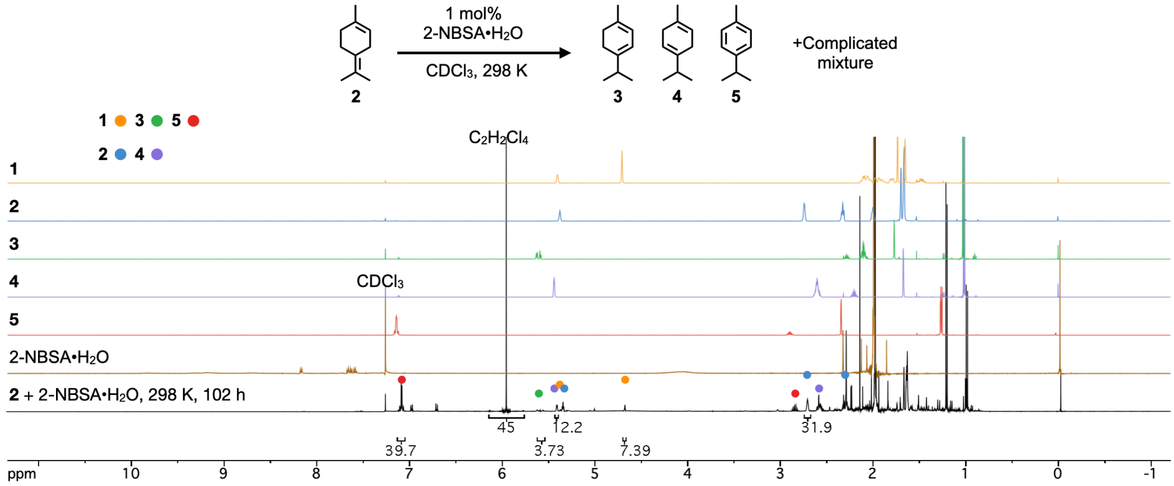

Fig. S20 ^{1}H NMR spectra (500 MHz, $CDCl_3$, 300 K) of **1**–**5** and the reaction mixture of terpinolene (**2**) and 2-NBSA·H_2O in $CDCl_3$ at 298 K after 102 h.

5. Crystal structures of MMF soaked in a solution of terpenes

5.1 Crystal structure of (+)-limonene@MMF

MMF crystals (ca. 0.5 mg) were soaked in a $CHCl_3$ solution of (+)-limonene (**1**) (1.0 M, 100 µL, 100 µmol) at 298 K for 24 h in a capped microtube. One MMF crystal was then picked up and immediately mixed with paratone oil and single-crystal XRD was measured at 93 K.

Crystal data for (+)-limonene@MMF

Crystal data for $(Pd_3\mathbf{L}Cl_6)_4$·((+)-limonene)$_{0.6}$·$(H_2O)_{4.5}$: $C_{174}H_{177.6}Cl_{24}N_{24}O_{4.5}Pd_{12}$, Fw = 4804.03, crystal dimensions 0.08 × 0.10 × 0.11 mm^3, monoclinic, space group $P2_1$, a = 14.2647(1), b = 52.2312(10), c = 19.5113(3) Å, β = 91.295(1)°, V = 14533.4(4) Å^3, Z = 2, ρ_{calcd} = 1.098 g cm^{-3}, μ = 8.17 mm^{-1}, T = 93 K, λ(CuKα) = 1.54187 Å, $2\theta_{max}$ = 147.3°, 160911/56440 reflections collected/unique (R_{int} = 0.0658), R_1 = 0.1006 ($I > 2\sigma(I)$), wR_2 = 0.3087 (for all data), GOF = 1.095, largest diff. peak and hole 3.340/–1.950 eÅ^{-3}, Flack parameter = 0.245(15), Hooft parameter = –0.061(8). CCDC deposit number 2133386. Several restraints were applied to the MMF and the guest molecules to prevent the structure from collapsing during the least-squares refinement. The structure was refined as an inversion twin.

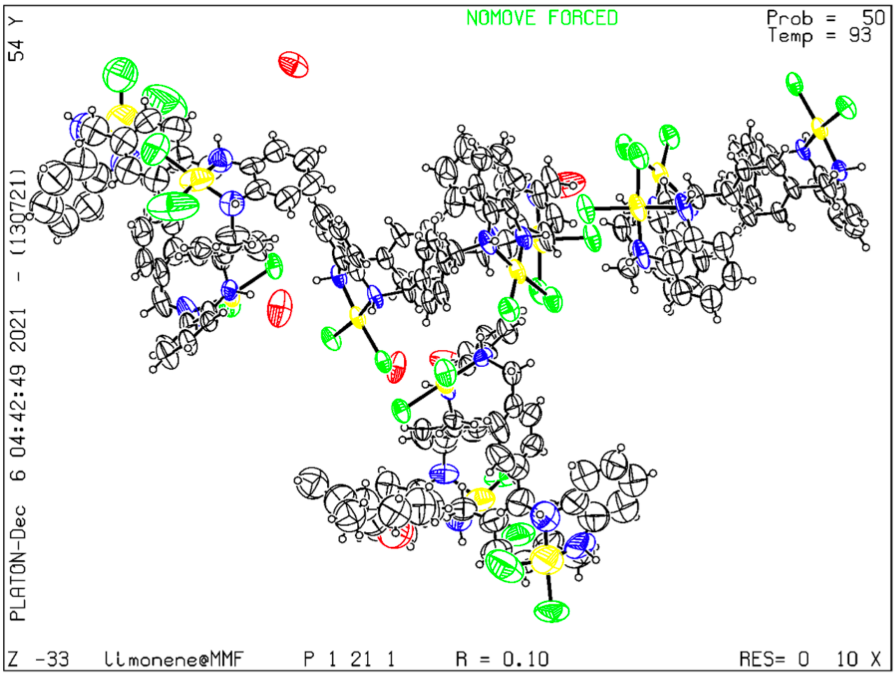

Fig. S21 ORTEP drawing of (+)-limonene@MMF at the 50% probability level. Color: C black, N blue, O red, Cl green and Pd yellow.

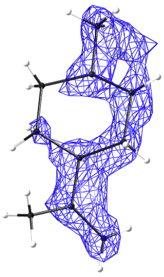

Fig. S22 Electron density maps of (+)-limonene in the crystal structure of (+)-limonene@MMF (contour level: 1.20 e^{-}/Å^{3}).

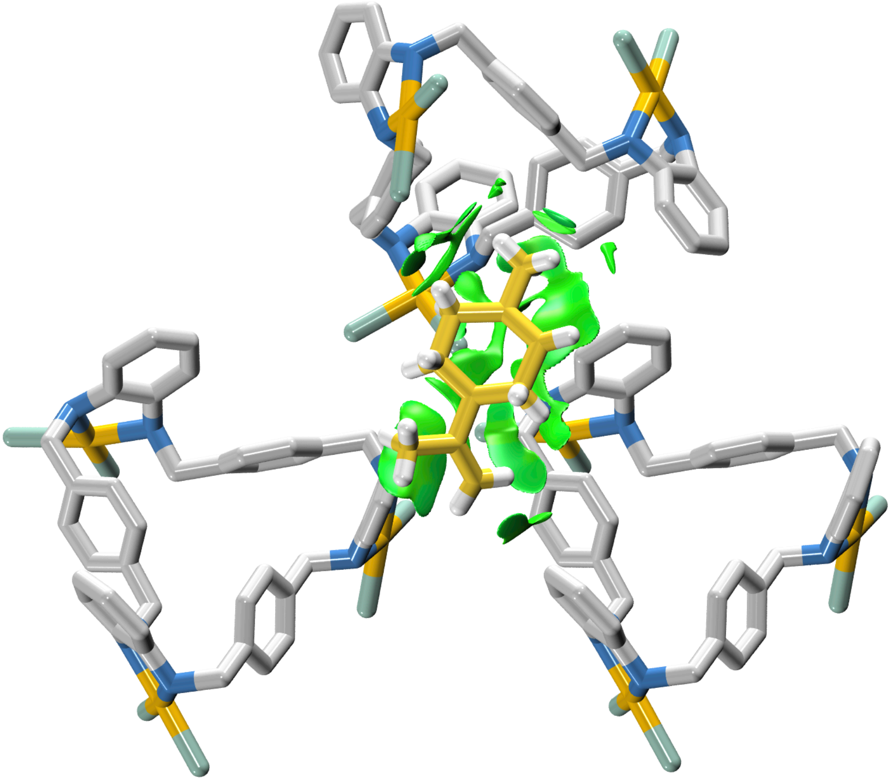

Fig. S23 Plot of noncovalent interactions between (+)-limonene (**1**) and three adjacent macrocycles. Noncovalent interactions: green surface (s = 0.5 a.u., ρ < 0.05 a.u.). Macrocycles and (+)-limonene: sticks model. MMF: Pd, yellow; Cl, green; N, blue; C, grey. **1**: C, yellow; H, white. Hydrogen atoms attached to macrocycles were omitted for clarity.

5.2 Crystal structure of terpinolene@MMF

MMF crystals (ca. 0.5 mg) were soaked in a $CHCl_3$ solution of terpinolene (**2**) (1.0 M, 100 µL, 100 µmol) at 298 K for 24 h in a capped micro-tube. One MMF crystal was then picked up and immediately mixed with paratone oil and single-crystal XRD was measured at 93 K.

Crystal data for terpinolene@MMF

Crystal data for $(Pd_3\mathbf{L}Cl_6)_2\cdot(CH_3CN)\cdot(CHCl_3)_{3.3}\cdot(H_2O)_2$: $C_{89.3}H_{90.3}Cl_{21.8}N_{13}O_2Pd_6$, Fw = 2786.43, crystal dimensions 0.10 × 0.10 × 0.20 mm^3, monoclinic, space group $P2_1/c$, a = 19.4857(2), b = 51.8380(7), c = 14.3167(1) Å, β = 90.706(1)°, V = 14460.2(3) Å^3, Z = 4, ρ_{calcd} = 1.280 g cm^{-3}, μ = 9.90 mm^{-1}, T = 93 K, λ(CuKα) = 1.54187 Å, $2\theta_{max}$ = 146.8°, 175959/28222 reflections collected/unique (R_{int} = 0.0971), R_1 = 0.1322 ($I > 2\sigma(I)$), wR_2 = 0.3637 (for all data), GOF = 1.084, largest diff. peak and hole 3.140/–1.950 eÅ^{-3}. CCDC deposit number 2133390. Several restraints were applied to the MMF and the guest molecules to prevent the structure from collapsing during the least-squares refinement.

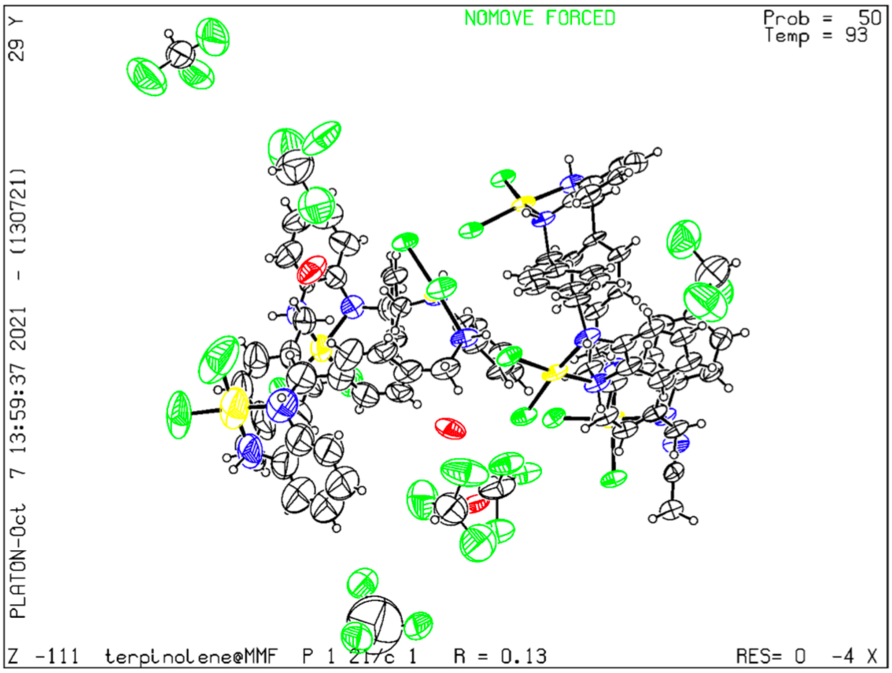

Fig. S24 ORTEP drawing of terpinolene@MMF at the 50% probability level. Color: C black, N blue, O red, Cl green and Pd yellow.

5.3 Crystal structure of (–)-β-pinene@MMF

MMF crystals (ca. 0.5 mg) were soaked in a CH_3CN solution of (–)-β-pinene (**7**) (1.0 M, 100 μL, 100 μmol) at 298 K for 24 h in a capped micro-tube. One MMF crystal was then picked up and immediately mixed with paratone oil and single-crystal XRD was measured at 93 K.

Crystal data for (–)-β-pinene @MMF

Crystal data for $(Pd_3\mathbf{L}Cl_6)_4\cdot$((–)-β-pinene)$_{0.91}\cdot(CH_3CN)\cdot(H_2O)_2$: $C_{179.1}H_{185.6}Cl_{24}N_{25}O_2Pd_{12}$, Fw = 4847.88, crystal dimensions 0.01 × 0.02× 0.05 mm^3, monoclinic, space group $P2_1$, a = 14.3205(1), b = 50.1973(12), c = 19.4935(3) Å, β = 91.679(1)°, V = 14006.9(4) Å^3, Z = 2, ρ_{calcd} = 1.149 g cm^{-3}, μ = 8.47 mm^{-1}, T = 93 K, λ(CuKα) = 1.54187 Å, $2\theta_{max}$ = 145.1°, 147264/53297 reflections collected/unique (R_{int} = 0.0849), R_1 = 0.1752 ($I > 2\sigma(I)$), wR_2 = 0.4764 (for all data), GOF = 1.795, largest diff. peak and hole 4.250/–3.050 eÅ^{-3}, Flack parameter = 0.23(3), Hooft parameter = –0.048(8). CCDC deposit number 2133389. Several restraints were applied to the MMF and the guest molecules to prevent the structure from collapsing during the least-squares refinement. The structure was refined as an inversion twin.

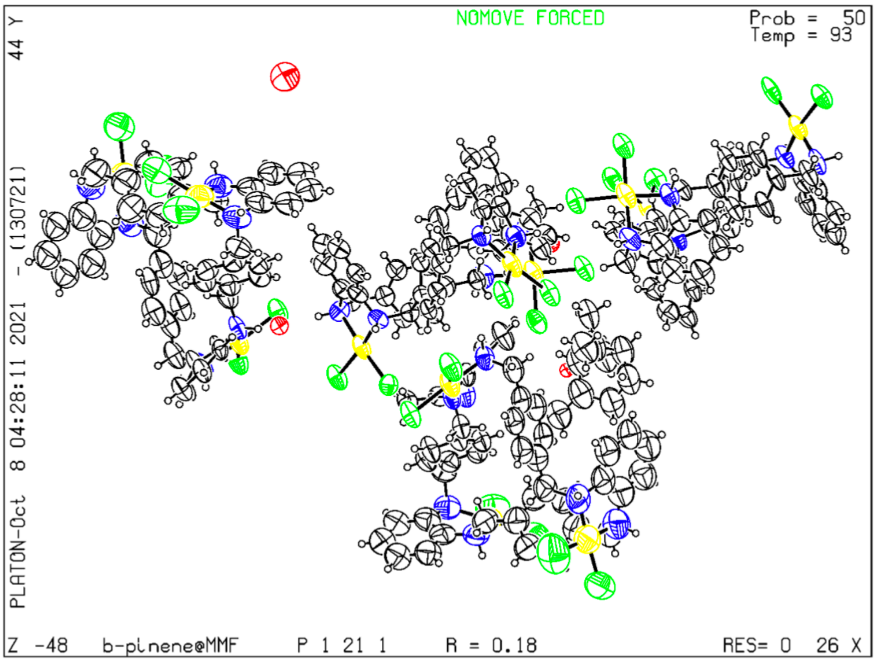

Fig. S25 ORTEP drawing of (–)-β-pinene@MMF at the 50% probability level. Color: C black, N blue, O red, Cl green and Pd yellow.

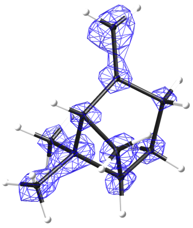

Fig. S26 Electron density maps of (–)-β-pinene in the crystal structure of (–)-β-pinene@MMF (contour level: 1.80 $e^{-}/Å^{3}$).

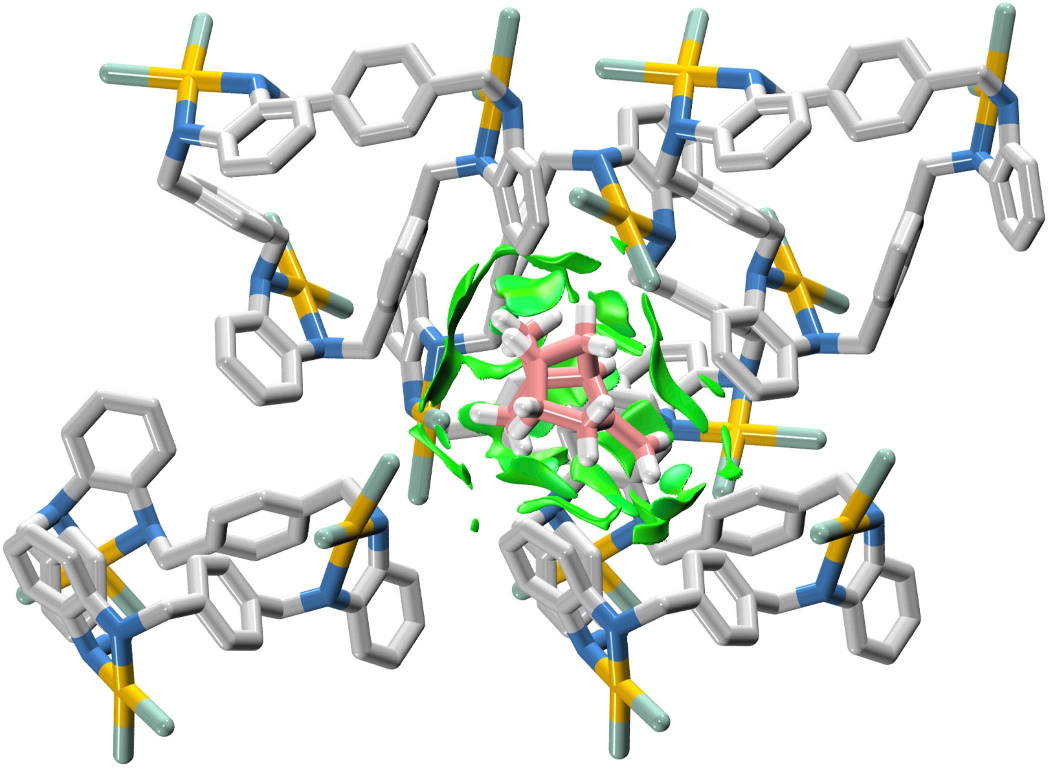

Fig. S27 Plot of non-covalent interactions between (–)-β-pinene (**7**) and five adjacent macrocycles. Non-covalent interactions: green surface (s = 0.5 a.u., ρ < 0.05 a.u.). Macrocycles and (–)-β-pinene: sticks model. MMF: Pd, yellow; Cl, green; N, blue; C, grey. **7**: C, pink; H, white. Hydrogen atoms attached to macrocycles were omitted for clarity.

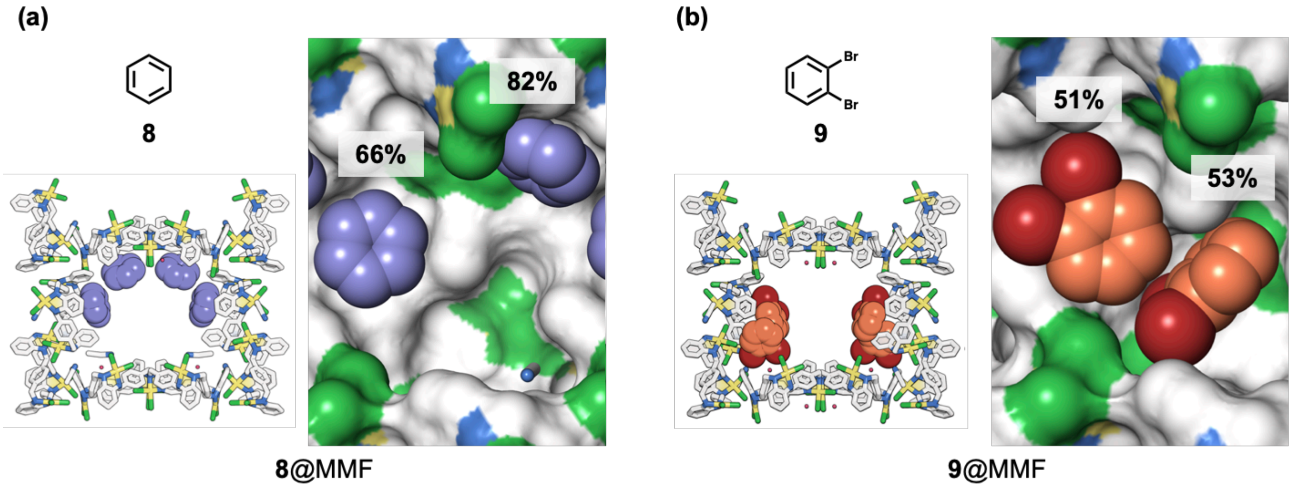

Fig. S28 Crystal structures of MMF soaked in an acetonitrile solution of (a) benzene (**8**) and (b) 1,2-dibromobenzene (**9**).[1] These binding structures are for reference only, because the soaking solvent (acetonitrile) is different from the reaction solvent (chloroform). MMF: sticks model or surface model; **8** and **9**: space-filling mode; water: sticks model. MMF: Pd, yellow; Cl, green; N, blue; C, grey. **8**: C, purple. **9**: C, orange; O, red. Water: O, red. Hydrogen atoms attached to MMF were omitted for clarity.

Green and blue surface represents exposed Cl and N-H groups of MMF, respectively. The molecular occupancies of **8** and **9** were denoted.

6. Cyclization of nerol using 2-NBSA@MMF and 2-NBSA·H_2O

6.1 Procedure for the cyclization of nerol using 2-NBSA@MMF

Crystals of 2-NBSA@MMF (ca. 0.5 mg, 0.1 µmol of 2-NBSA in MMF, 1 mol%) immediately after preparation and a $CDCl_3$ solution of nerol (10 mM, 1.0 mL, 10 µmol) were set in an NMR tube. This heterogeneous mixture was shaken on a shaker at 298 K and the time-course of the reaction was monitored by ^{1}H NMR measurements (500 MHz, $CDCl_3$, 300 K). The conversion ratios of nerol (**10**) and the yields of the main products were evaluated from the internal standard of 1,1,2,2-tetrachloroethane (6 mM, 1.0 mL, 6 µmol). Treatment of nerol (**10**) with 2-NBSA@MMF resulted in 99% conversion of nerol (**10**) to limonene (45%), terpinolene (**2**) (34%) and *p*-cymene (**5**) (4%) at 298 K after 100 h.

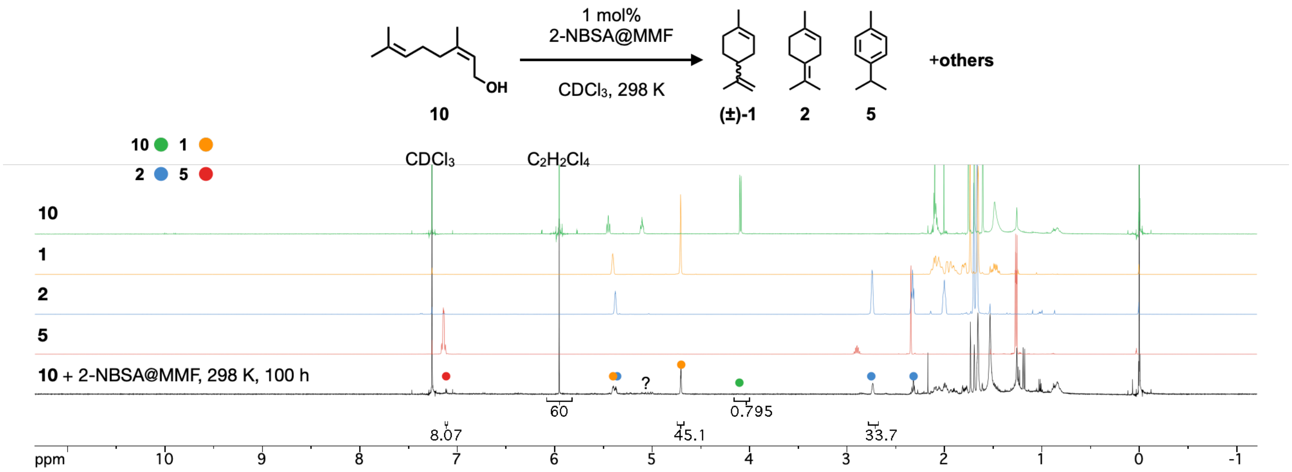

Fig. S29 ^{1}H NMR spectra (500 MHz, $CDCl_3$, 300 K) of **10, 1, 2, 5**, and the reaction mixture of nerol **(10)** and 2-NBSA@MMF in $CDCl_3$ at 298 K after 100 h.

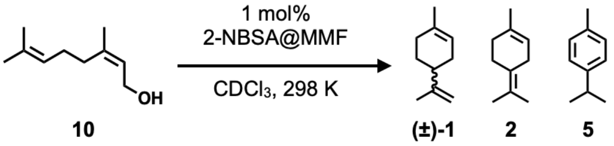

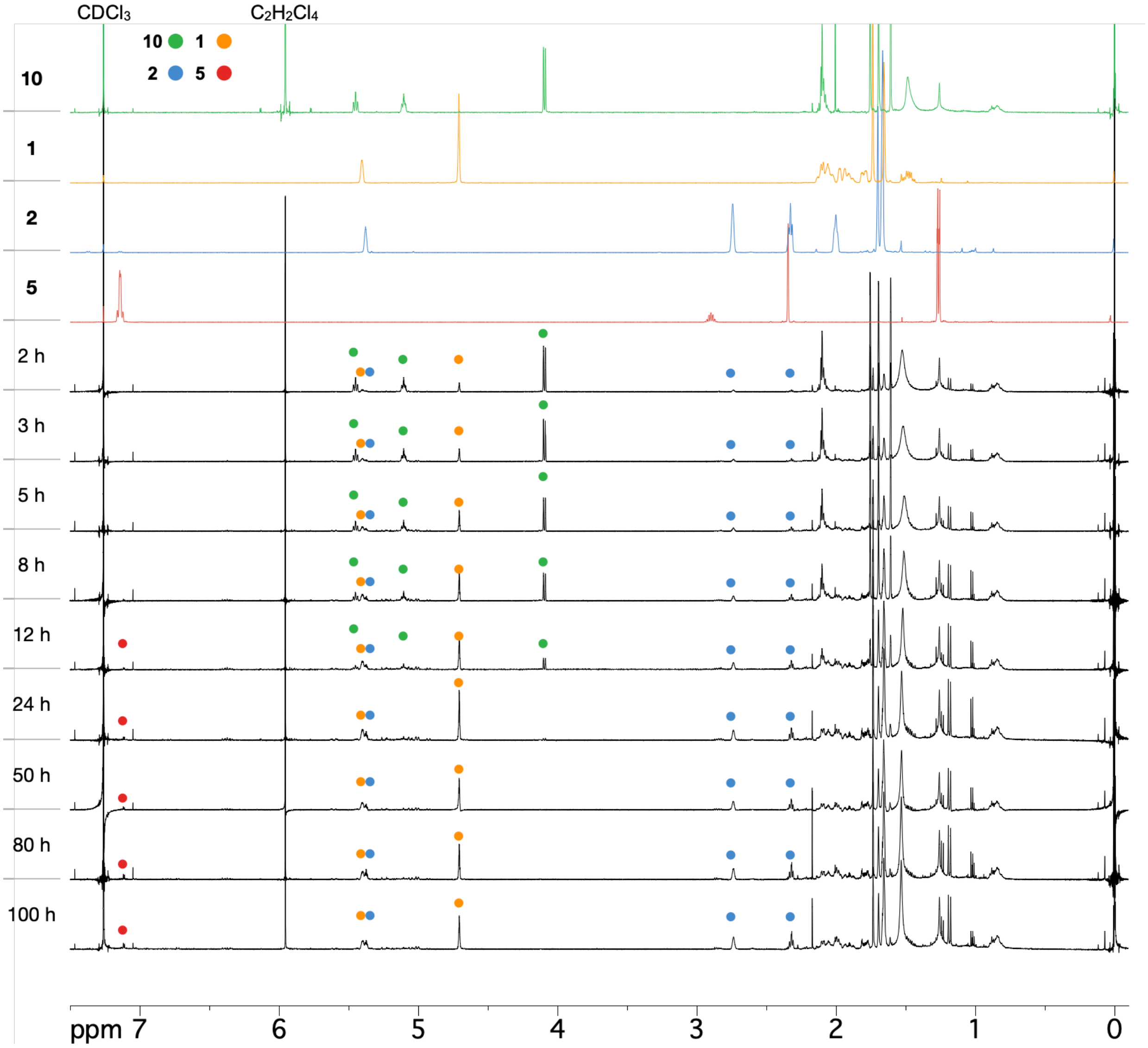

Fig. S30 ^{1}H NMR spectra (500 MHz, $CDCl_3$, 300 K) of **10, 1, 2, 5**, and the time-course analysis of the reaction mixture of nerol (**10**) and 2-NBSA@MMF in $CDCl_3$ at 298 K.

6.2 Procedure for the isomerization of nerol using 2-NBSA·H_2O

A $CDCl_3$ solution of 2-NBSA·H_2O (1 mM, 0.1 mL, 0.1 µmol, 1 mol%) and a $CDCl_3$ solution of nerol (11 mM, 0.9 mL, 9.9 µmol) were set in an NMR tube. This mixture was shaken on a shaker at 298 K and the time-course of the reaction was monitored by ^{1}H NMR measurements (500 MHz, $CDCl_3$, 300 K). The conversion ratios of nerol (**10**) and the yields of the main products were evaluated from the internal standard of 1,1,2,2-tetrachloroethane (4 mM, 1.0 mL, 4 µmol). Treatment of nerol (**10**) with 2-NBSA@MMF resulted in 100% conversion of nerol (**10**) to limonene (4%), terpinolene (**2**) (13%) and *p*-cymene (**5**) (30%) at 298 K after 100 h.

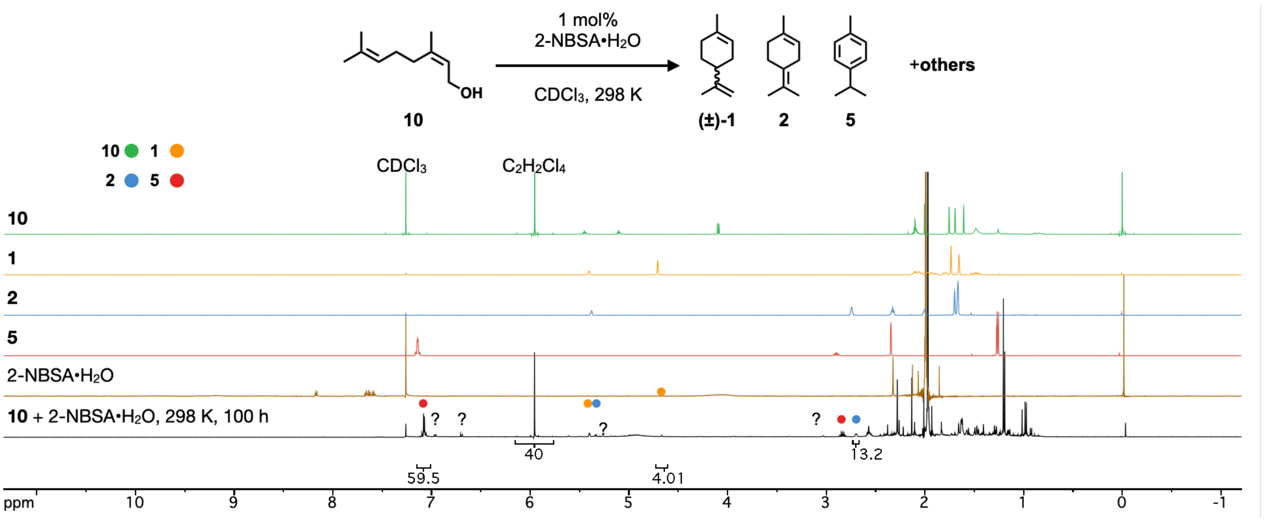

Fig. S31 ^{1}H NMR spectra (500 MHz, $CDCl_3$, 300 K) for **10, 1, 2**, **5**, 2-NBSA·H_2O and the reaction mixture of nerol (**10)** and 2-NBSA·H_2O in $CDCl_3$ at 298 K after 100 h.

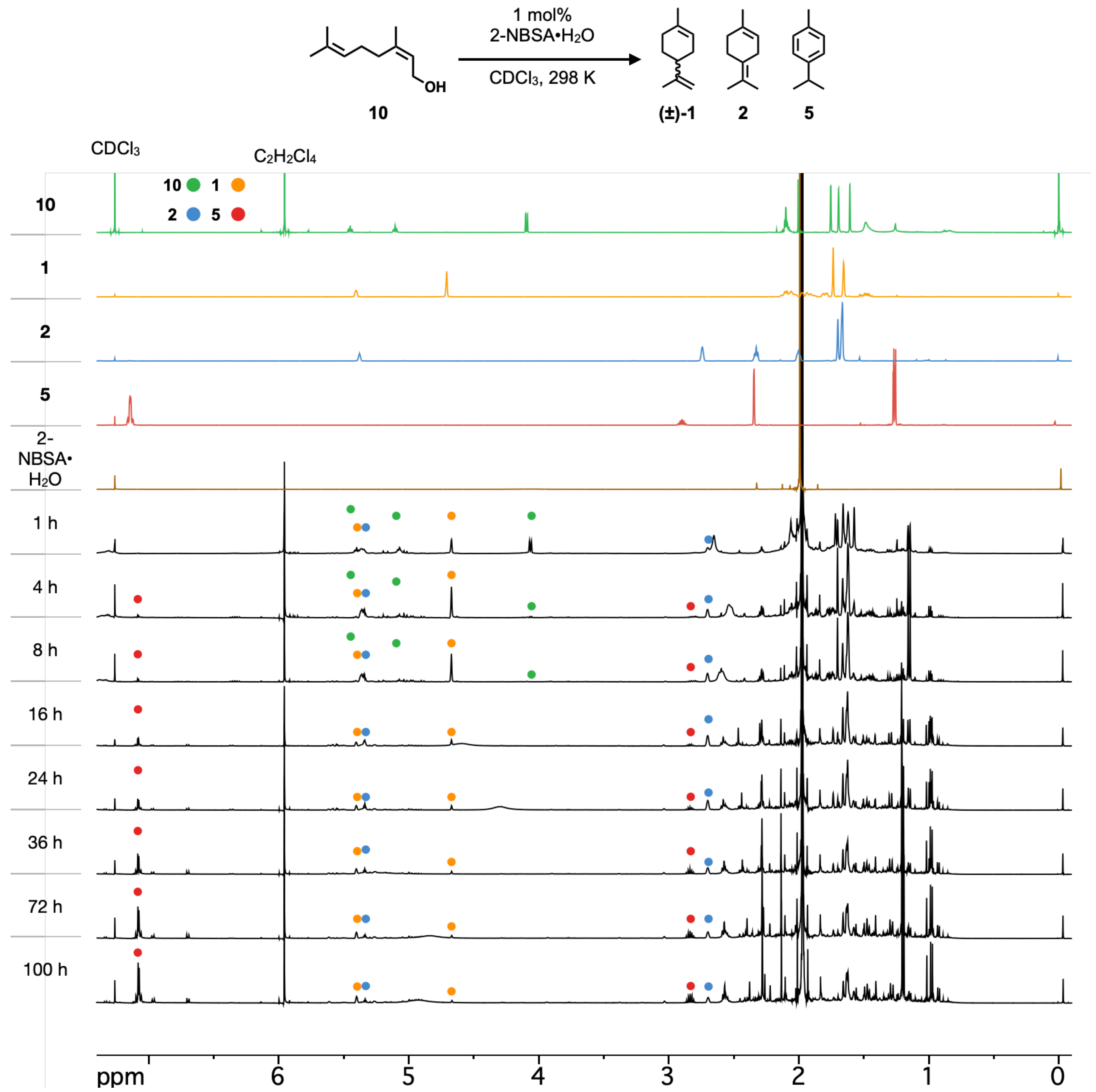

Fig. S32 ^{1}H NMR spectra (500 MHz, $CDCl_3$, 300 K) of **10, 1, 2, 5** and 2-NBSA·H_2O, and the time-course analysis of the reaction mixture of nerol (**10**) and 2-NBSA·H_2O in $CDCl_3$ at 298 K.

7. References

1. S. Tashiro, R. Kubota and M. Shionoya, *J. Am. Chem. Soc.*, 2012, **134**, 2461–2464.
2. S. Tashiro, H. Yonezawa, R. Kubota, T. Umeki and M. Shionoya, *Chem. Commun.*, 2016, **52**, 7657–7660.
3. O. V. Dolomanov, L. J. Bourhis, R. J. Gildea, J. A. Howard and H. Puschmann, *J. Appl. Crystallogr.*, 2009, **42**, 339–341.
4. G. M. Sheldrick, *Acta Crystallogr. C*, 2015, **71**, 3–8.
5. C. B. Hübschle, G. M. Sheldrick and B. Dittrich, *J. Appl. Crystallogr.*, 2011, **44**, 1281–1284.
6. E. R. Johnson, S. Keinan, P. Mori-Sánchez, J. Contreras-García, A. J. Cohen and W. Yang, *J. Am. Chem. Soc.*, 2010, **132**, 6498–6506.
7. R. Chaudret, B. De Courcy, J. Contreras-García, E. Gloaguen, A. Zehnacker-Rentien, M. Mons and J. P. Piquemal, *Phys. Chem. Chem. Phys.*, 2014, **16**, 9876.
8. J. Contreras-García, E. R. Johnson, S. Keinan, R. Chaudret, J.-P. Piquemal, D. N. Beratan and W. Yang, *J. Chem. Theory and Comput.*, 2011, **7**, 625–632.
9. W. Humphrey, A. Dalke and K. Schulten, *J. Mol. Graph.*, 1996, **14**, 33–38.
